# Anti-Inflammatory and Antimicrobial Volatile Oils: Fennel and Cumin Inhibit Neutrophilic Inflammation via Regulating Calcium and MAPKs

**DOI:** 10.3389/fphar.2021.674095

**Published:** 2021-10-11

**Authors:** Michal Korinek, Heba Handoussa, Yi-Hong Tsai, You-Ying Chen, Meng-Hua Chen, Zan-Wei Chiou, Yu Fang, Fang-Rong Chang, Chia-Hung Yen, Chung-Fan Hsieh, Bing-Hung Chen, Mohamed El-Shazly, Tsong-Long Hwang

**Affiliations:** ^1^ Graduate Institute of Natural Products, College of Pharmacy, Kaohsiung Medical University, Kaohsiung, Taiwan; ^2^ Graduate Institute of Natural Products, College of Medicine, Chang Gung University, Taoyuan, Taiwan; ^3^ Department of Biotechnology, College of Life Science, Kaohsiung Medical University, Kaohsiung, Taiwan; ^4^ Department of Pharmaceutical Biology, Faculty of Pharmacy and Biotechnology, German University in Cairo, Cairo, Egypt; ^5^ Department of Marine Biotechnology and Resources, National Sun Yat-sen University, Kaohsiung, Taiwan; ^6^ The Research Center for Emerging Viral Infections, College of Medicine, Chang Gung University, Taoyuan, Taiwan; ^7^ Department of Pharmacognosy, Faculty of Pharmacy, Ain-Shams University, Cairo, Egypt; ^8^ Research Center for Chinese Herbal Medicine, Research Center for Food and Cosmetic Safety, Graduate Institute of Health Industry Technology, College of Human Ecology, Chang Gung University of Science and Technology, Taoyuan, Taiwan; ^9^ Department of Anesthesiology, Chang Gung Memorial Hospital, Taoyuan, Taiwan; ^10^ Department of Chemical Engineering, Ming Chi University of Technology, New Taipei City, Taiwan

**Keywords:** fennel, cumin, anti-inflammatory activity, respiratory burst, degranulation, formyl peptide receptor, antimicrobial activity, essential oil

## Abstract

Neutrophilic inflammatory diseases, such as chronic obstructive pulmonary disease (COPD), acute respiratory distress syndrome (ARDS), or psoriasis, exert a huge burden on the global health system due to the lack of safe and effective treatments. Volatile oils from terrestrial plants showed impressive therapeutic effects against disorders of the skin, digestive system, lungs, liver, metabolism, and nervous system. However, their effect on the immune system and neutrophil function is still elusive. Fennel, cumin, marjoram, lavender, caraway, and anise are the common nutraceuticals that are widely used in the Mediterranean diet. The volatile oils of these herbs were screened for various biological activities, including anti-inflammatory, anti-allergic, antimicrobial, and antiviral effects. Several oils showed anti-inflammatory and antimicrobial potential. Fennel (*Foeniculum vulgare*) and cumin (*Cuminum cyminum*) fruits' volatile oils significantly suppressed the activation of human neutrophils, including respiratory burst and the degranulation induced by formyl peptide receptor agonists fMLF/CB and MMK1 in the human neutrophils (IC_50_, 3.8–17.2 µg/ml). The cytotoxic effect and free-radical scavenging effects (ABTS, DPPH) of these oils did not account for the observed effects. Both fennel and cumin volatile oils significantly shortened calcium influx recovery time and inhibited phosphorylation of mitogen-activated protein kinases (p38, JNK, and ERK) expression. The gas chromatography–mass spectrometry analysis of these oils revealed the presence of estragole and cuminaldehyde as the major components of fennel and cumin volatile oils, respectively. Our findings suggested that cumin and fennel, common in the Mediterranean diet, hold the potential to be applied for the treatment of neutrophilic inflammatory diseases.

**GRAPHICAL ABSTRACT:**
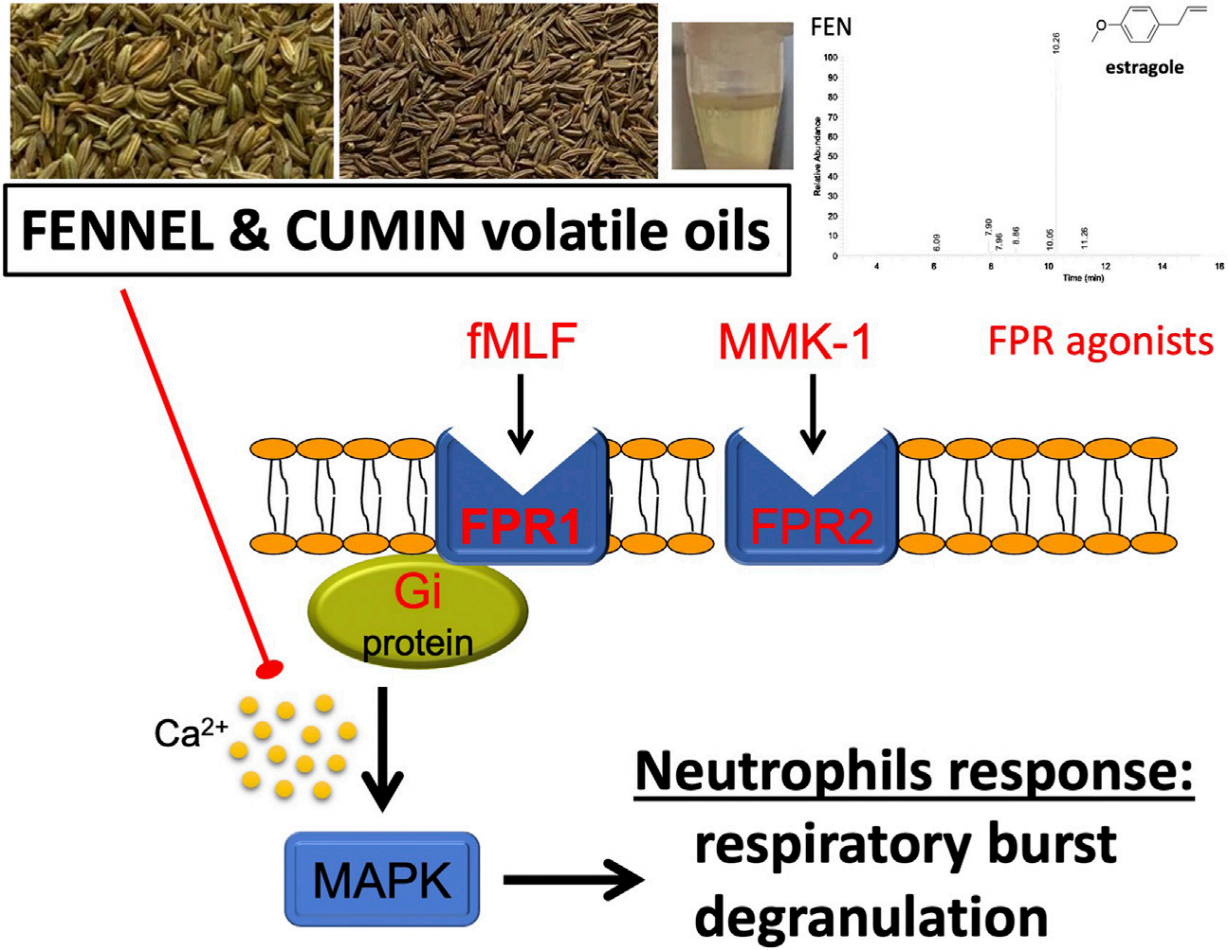
A summary of the study, volatile oils inhibit neutrophilic inflammation.

## Introduction

The World Health Organization reported that more than three billion human beings depend on traditional remedies, such as herbal drugs and related products, for their primary health care. Volatile or essential oils are usually referred to as mixtures of volatile hydrocarbons and their oxygenated derivatives with a characteristic scent. Volatile oils are produced by certain organs of aromatic plants (buds, flowers, leaves, stems, branches, seeds, berries, roots, wood, or bark) and stored in secretory cells or cavities (Bakkali et al., 2008). Volatile oils are not only popular and widely used in the perfume industry, but many of them are also generally recognized as safe and used in therapeutics and the food industry. Many preclinical studies documented the antitumor, anti-inflammatory, antioxidant, and antibacterial properties of volatile oils in several animal models and bioassays (De, 2004; Edris, 2007; Saviuc et al., 2015; Sharifi-Rad et al., 2017).

Volatile oils are known for their antioxidant activity due to their free radical scavenging ability. This activity supports their use in food preservation and for the management of many ailments, such as cancers, and neurodegenerative, cardiovascular, and immune system diseases (Pourmortazavi and Hajimirsadeghi, 2007). Volatile oils possess significant antiseptic, antibacterial, antiviral, anti-parasitic, antifungal, and insecticidal activities (Burt, 2004). Therefore, volatile oils are widely used as antimicrobial agents with a high safety index and potent activity against a wide array of pathogenic microorganisms.

Microbial infections can result in life-threatening conditions, resulting in an annual loss of millions of lives. Although penicillin discovery pushed back many aggressive pathogenic bacteria, certain strains evolved and developed powerful resistant mechanisms against most available antibiotics (Fischbach and Walsh, 2009). Natural products were always the backbone for the discovery of new antimicrobial agents. The antimicrobial activity of certain volatile oils has been documented in many studies (De et al., 1999; Hemaiswarya et al., 2008; Badgujar et al., 2014; Saviuc et al., 2015). However, published data are scarce on the antimicrobial and anti-inflammatory activities of some common volatile oils obtained from Egypt, such as cumin (*Cuminum cyminum*) and caraway (*Carum carvi*) oils.

Microbial infection stimulates inflammatory response, and the proper regulation of this response is crucial to maintain human body homeostasis and health. Inflammation is an innate immune response to a pathogenic invasion, infection, tissue injury, or other triggers. It is a complex biological response that includes the accumulation of blood and immune cells together with the release of inflammatory mediators affecting the structure and function of the tissue. Untreated acute inflammation can lead to chronic inflammation, which facilitates the development of other diseases, such as cancers and autoimmune, lung, and neurodegenerative diseases (Korkmaz et al., 2010). Human neutrophils serve as the first line of defense against pathogens and are important for the initiation of inflammatory reactions (Pérez-García et al., 2001). Human neutrophils, the most abundant innate immune cells, exert several mechanisms to destroy the invading microorganisms and activate other cells of the immune system (Tecchio and Cassatella, 2016). These offensive features include respiratory burst through the generation of large amounts of reactive oxygen species (ROS) such as superoxide, degranulation through the release of antimicrobial peptides and enzymes, such as elastase or myeloperoxidase, and neutrophil extracellular traps (NETs) formation. All these events contribute to the balanced response to infection (Nauseef and Borregaard, 2014). The uncontrolled inflammatory response may lead to the progression of many disorders such as chronic obstructive pulmonary disease (COPD), asthma (Wright et al., 2016), psoriasis, or cystic fibrosis (Korkmaz et al., 2010). Neutrophils also contribute to alveolar inflammation observed in acute respiratory distress syndrome (ARDS), a life-threatening complication associated with the current COVID-19 pandemic (Badraoui et al., 2020). It has been proposed that the elimination of elastase-related neutrophil proteases may reduce the progression of lung injury (Korkmaz et al., 2020). Also, neutrophils are overexpressed in the severe stages of coronavirus disease thus the suppression of their function may be beneficial in the coronavirus-associated ARDS (Chiang et al., 2020). Natural products have been the source of many promising anti-inflammatory agents (Saleem et al., 2018), but little is known on the function of volatile oils on human neutrophils.

Fennel, cumin, marjoram (syn. majoram), anise, caraway, and lavender are common herbs, and some of them are widely used in the Mediterranean diet. Volatile oils extracted from these herbs are used for rituals, perfumes (scent), aromatherapy, food, and pharmaceutical products; moreover, all except cumin are listed in the European and/or American Pharmacopoeia (Committee on Herbal Medicinal Products, 2016; Sharifi-Rad et al., 2017). Fennel and cumin volatile oils exhibit therapeutic effects against skin, digestive system, lungs, liver, metabolism, and nervous system disorders (Badgujar et al., 2014; Srinivasan, 2018; Singh et al., 2021). Despite their wide use, there is no complete understanding of the bioactivity effects of these volatile oils obtained from plants grown in certain Mediterranean countries such as Egypt. A plant's geographical source significantly affects its phytochemical content and bioactivity. Thus, our study aimed to fill the gap and investigate the effects of volatile oils on microbial infections and neutrophilic inflammation. Other biological activities of volatile oils were also evaluated, such as the anti-allergic, anti-viral against influenza H1N1 and enterovirus D68, antioxidant capacity expressed as NRF2 activity, and lipid formation effects. The chemical profile of the volatile oils was also investigated.

## Materials and Methods

### Reagents and Antibodies

The volatile oil samples were dissolved in dimethyl sulfoxide (DMSO) before running the experiments. Bovine serum albumin (BSA), Dulbecco's modified Eagle's medium (DMEM), fetal bovine serum (FBS), penicillin/streptomycin solution, and other cell culture reagents were purchased from Lonza (Basel, Switzerland). E10 medium consists of DMEM containing 10% FBS, 100 U/ml penicillin (Gibco, United States), 100 μg/ml streptomycin (Gibco, United States), 2 mM L-glutamine (Gibco, Brazil), and 0.1 mM nonessential amino acid mixture (Gibco, United States). Reduced glutathione (GSH), 5,5ʹ-dithiobis-2-nitrobenzoic acid (Ellman's reagent), thiobarbituric acid (TBA), trichloroacetic acid (TCA), genistein, dexamethasone, dextran, ferricytochrome *c*, cytochalasin B (CB), Triton X-100, *α*-tocopherol (vitamin E), 1,1-disphenyl-2-picryl-hydrazyl (DPPH), *N*-formyl-methionyl-leucyl-phenylalanine (fMLF), 2,2ʹ-azino-bis(3-ethylbenzothiazoline-6-sulfonic acid) (ABTS) diammonium salt, xanthine oxidase, DMSO, phorbol 12-myristate 13-acetate (PMA) were all purchased from Sigma-Aldrich (St. Louis, MO, United States). Ficoll^®^-Paque PLUS was purchased from GE Healthcare (Buckinghamshire, United Kingdom). Hank's balanced salt solution (HBSS) and DMEM were purchased from Gibco (Grand Island, NY, United States). Trypan blue was purchased from Biological Industries (Beit Haemek, Israel). Methoxysuccinyl-Ala-Ala-Pro-Val-*p*-nitroanilide was purchased from Calbiochem (La Jolla, CA, United States). MMK1 and LTB4 were purchased from Tocris Bioscience (Bristol, United Kingdom). IL8 was purchased from ProSpec (Ness-Ziona, Israel). Fluo-3 acetoxymethyl ester (Fluo-3/AM) was obtained from Molecular Probes (Eugene, OR, United States). Xanthine was purchased from Santa Cruz Biotechnology (Dallas, TX, United States). 2-(4-Iodophenyl)-3-(4-nitrophenyl)-5-(2,4-disulfophenyl)-2H-tetrazolium monosodium salt (water-soluble tetrazolium-1, WST-1) was purchased from Dojindo Laboratories (Kumamoto, Japan). Antibody for p38 mitogen-activated protein kinases (MAPK) (Cell Signaling Technology Cat# 9212, RRID:AB_330713), and phospho-p38 MAPK (Thr180/Tyr182) (Cell Signaling Technology Cat# 9211, RRID:AB_331641), SAPK/JNK (56G8) (Cell Signaling Technology Cat# 9258, RRID:AB_2141027), phospho-SAPK/JNK (Thr183/Tyr185) (Cell Signaling Technology Cat# 9251, RRID:AB_331659), ERK p44/42 MAPK (Erk1/2) (137F5) (Cell Signaling Technology Cat# 4695, RRID:AB_390779), and phospho-ERK p44/42 MAPK (Erk1/2) (Thr202/Tyr204) (Cell Signaling Technology Cat# 4370, RRID:AB_2315112) antibodies were purchased from Cell Signaling (Beverly, MA, United States). Secondary goat anti-rabbit IgG (H+L) antibody, horseradish peroxidase (HRP)-linked (Thermo Fisher Scientific Cat# 31460, RRID:AB_228341) was used for Western blotting experiments. All other chemicals were of analytical grade.

### Plant Material

Marjoram leaves (*Origanum majorana* L.), lavender aerial parts (*Lavandula angustifolia* Mill.), fennel fruits (*Foeniculum vulgare* Mill.), cumin fruits (*C. cyminum* L.), caraway fruits (*Carum carvi* L*.*), and anise fruits (*Pimpinella anisum* L.) were collected from the Orman Garden, Giza region, Egypt. The plants were kindly authenticated by the agricultural engineer Therese Labib, a consultant at Orman Botanical Garden, Giza, and National Gene Bank at the Ministry of Agriculture. Voucher specimens of the authenticated leaves were deposited at the Department of Pharmaceutical Biology, Faculty of Pharmacy and Biotechnology, German University in Cairo University, Cairo, Egypt (PHB-PHB-GUC-OM-L-23-MAR-2020, PHB-PHB-GUC-LO-AP-23-MAR-2020, PHB-PHB-GUC-FV-F-23-MAR-2020, PHB-PHB-GUC-CCY-F-23-MAR-2020, PHB-PHB-GUC-CCA-F-23-MAR-2020, and PHB-PHB-GUC-PA-L-23-MAR-2020). Volatile oils were obtained from the intact fresh leaves and fruits and kept at 20–25°C to be used within 5 h after collection.

### Volatile Oils Preparation

Fresh intact parts (800 g) of each plant were hydrodistilled in a modified Karlsruher apparatus using *n*-hexane as the collecting solvent until there was no significant increase in the volume of each oil within 4 h. Each oil was passed through a filter paper containing anhydrous sodium sulfate to entrap moisture. Oils were stored in glass bottles in the dark at 4°C in the refrigerator.

### *In vitro* Assessment of Antimicrobial Activity

#### Bacterial Strains

The bacterial strains were obtained from the American Type Culture Collection (ATCC) standard strains (*Staphylococcus aureus* ATCC 12600, *Escherichia coli* ATCC 11775, *Candida albicans* ATCC 90028, *Vibrio harveyi* ATCC 25919, *Pseudomonas aeruginosa* ATCC 31156, and *Bacillus subtilis* ATCC 6633) and stored at −80°C. Before use, the strains were cultured. *C. albicans* and *V. harveyi* were cultured in Marine Broth (MA) at 25°C for 24 h. *P. aeruginosa*, *E. coli*, *B. subtilis*, and *S*. *aureus* were cultured in Mueller–Hinton broth (MH) at 37°C for 16–18 h.

#### Agar Disk-Diffusion Assay

The antimicrobial activity of the volatile oils was determined by the agar disk-diffusion assay according to a previously reported method with some modifications (Balouiri et al., 2016). The agar disk-diffusion assay was used to determine the antiproliferative activity of the tested compounds against the fungus *C. albicans*; gram-positive bacterial strains *S. aureus* and *B. subtilis*; and gram-negative strains *E. coli*, *V. harveyi*, and *P. aeruginosa*. The pathogenic strains were inoculated in a culture tube containing MH or MA, and the density of the bacterial suspension was adjusted to the 0.5 McFarland standards. A sterile cotton swab was used to spread the bacterial suspension onto the agar plates. Filter paper disks (about 6 mm in diameter) including the tested sample were placed onto the surface of plates inoculated with the pathogenic strains. Plates were then incubated at optimum temperatures (25°C for up to 24 h and 37°C for 16–18 h). The antimicrobial activity was determined by the presence of a clear zone of inhibition around the disk, and the diameter of the inhibition zone was measured.

#### Measurements of the Minimum Inhibitory Concentration

According to the above inoculated pathogenic strains method which was performed in 96 flat-bottom microtiter plates following the microdilution method, each volatile oil was diluted in a microtiter plate well using equivalent concentrations of 0.025, 0.05, 0.1, 0.2, 0.5, 0.8, 1.0, 1.5, 2.0, 2.5, and 3.0% v/v. An inoculum volume of 1 × 10^6^ CFU/ml was used in each microtiter plate well. Streptomycin and penicillin G (positive controls) and the medium (negative control) were employed under comparable experimental conditions. Values conversion from % v/v to mg/ml using the density values of each oil and calculation of minimal inhibition concentrations (MIC)—MIC50% and MIC90%—for each tested bacterial strain were performed. To assess the microbial growth, optical densities were read at 600 nm (OD_600_) using a microplate reader. Calculation of MIC was performed up to 512 μg/ml according to the literature (Rezzoug et al., 2019).

### *In vitro* Assessment of the Anti-inflammatory Activity

#### Preparation of Human Neutrophils

Blood was taken from healthy human donors (20–30 years old) by venipuncture using a protocol approved by the institutional review board at the Chang Gung Memorial Hospital. Neutrophils were isolated using a standard method as previously described (Yang et al., 2013). In brief, the blood samples were processed by dextran sedimentation and Ficoll-Paque centrifugation followed by hypotonic lysis of contaminated red blood cells (Boyum et al., 1991). The segregated neutrophils were suspended and stored in pH 7.4 Ca^2+^-free HBSS at 4°C before the experiments. Wright-Giemsa stain was applied to confirm the purity of the neutrophil suspension. The viability of >98% was confirmed by the trypan blue exclusion method.

#### Measurement of Superoxide Generation

Superoxide dismutase (SOD)–inhibitable ferricytochrome *c*, which can be reduced by superoxide, was utilized to evaluate the superoxide release in activated human neutrophils (Babior et al., 1973). The method was described in a previous study (Chen et al., 2014). Human neutrophils (6 × 10^5^ cells/ml) were incubated in HBSS containing ferricytochrome *c* (0.6 mg/ml) and CaCl_2_ (1 mM) and equilibrated at 37°C for 2 min and then incubated with the volatile oils or DMSO (control) for 5 min. Cells were primed by CB (1 μg/ml) for 3 min and then activated with fMLF (FPR1 agonist, 100 nM), MMK1 (FPR2 agonist, 300 nM) or DMSO (control) for 10 min, or unprimed cells treated with PMA (protein kinase C activator, 10 nM, 3 × 10^5^ cells/ml) for 12 min. The absorbance was continuously monitored at 550 nm using a double-beam, six-cell positioner spectrophotometer Hitachi U-3010 with constant stirring (Hitachi Inc., Tokyo, Japan). Calculations were based on the differences in absorbance in the presence or absence of SOD (100 U/ml) divided by the extinction coefficient for ferricytochrome *c*–reduced form (*ε* = 21.1/mM/10 mm). Genistein was used as the positive control.

#### Measurement of Elastase Release

Elastase release was measured by the degranulation of azurophilic granules in human neutrophils (Hwang et al., 2006a). Human neutrophils (6 × 10^5^ cells/ml) were equilibrated with elastase substrate MeO-Suc-Ala-Ala-Pro-Val-*p*-nitroanilide (100 μM) in HBSS supplemented with CaCl_2_ (1 mM) at 37°C for 2 min and then incubated with volatile oil samples or DMSO (control) for 5 min. Human neutrophils were then activated by 100 nM fMLF, or 300 nM MMK1, and 100 nM leukotriene B4 (a BLT1 receptor agonist, 100 nM) in the presence of 0.5 μg/ml CB, or with 100 ng/ml interleukin 8 (IL-8) in the presence of 2 μg/ml CB for 10 min. Changes in absorbance at 405 nm were continuously monitored by using a spectrometer (Hitachi U-3010, Tokyo, Japan) to record the elastase release. The results are expressed as the percent of the initial rate of elastase release in the fMLF/CB-activated, drug-free control system. Genistein was used as the positive control.

#### Evaluation of Lactated Dehydrogenase Release

To evaluate the possible cytotoxicity of the tested samples, lactate dehydrogenase (LDH) release was determined using a commercially available LDH assay kit (Promega, Madison, WI) according to the manufacturer's instructions (Liu et al., 2019). Neutrophils (6 × 10^5^/ml) were equilibrated at 37°C for 2 min and treated with the tested samples for 15 min. They were centrifuged at 200 *g* at 4°C for 8 min. LDH assay reagents were added to the supernatant, the cells were incubated for 30 min in the dark, and the fluorescence was measured. Cytotoxicity was represented by LDH release in treated cells, or a cell-free medium compared with a percentage of the total LDH released. The total LDH released was determined by lysing cells with 0.1% Triton X-100 for 30 min at 37°C.

#### Determination of Elastase Enzymatic Activity

The samples were evaluated and tested for direct effects on elastase enzymatic activity (Korinek et al., 2021). Briefly, the neutrophil suspension (6 × 10^5^ cells/ml) was preheated for 5 min in the presence of CaCl_2_ (1 mM) at 37°C. Priming agent CB (1.5 μg/ml) was added for 2 min, followed by fMLF (0.1 μM) for 15 min, and the cells were centrifuged at 1,000 g for 5 min at 4°C. Then, the supernatant containing elastase was preheated at 37°C for 5 min, and the volatile oil samples were added. Finally, 0.1 mM of the substrate methoxysuccinyl-Ala-Ala-Pro-Val-*p*-nitroanilide was added for 10 min, followed by the measurement of absorbance at 405 nm.

#### Determination of Intracellular Calcium Concentration ([Ca^2+^]i)

For measurement of intracellular calcium (Hwang et al., 2009), neutrophils (6 × 10^5^ cells/ml) were incubated with Fluo-3/AM (2 μM) at 37°C for 30 min. The supernatant was removed after centrifugation at 200 *g* at 4°C for 8 min. The resuspended cells in HBSS stained with Fluo-3/AM were treated with fennel (FEN) and cumin (CUMIN) volatile oils (10–30 μg/ml) for 5 min in the presence of 1 mM CaCl_2_ at 37°C and stimulated by 0.1 μM fMLF. The fluorescence changes were continuously monitored by a Hitachi F-4500 spectrofluorometer (Tokyo, Japan) with constant stirring at 37°C in a quartz cuvette. The excitation and emission wavelengths were set to 488 and 520 nm, respectively. Finally, 0.05% Triton X-100 was added to obtain the maximum fluorescence value (F_max_), and 20 mM EGTA was added to obtain the minimum fluorescence value (F_min_). The change in intracellular Ca^2+^ concentration was calculated according to the following formula: [Ca^2+^]_i_ = K_d_ × [(F − F_min_)/(F_max_ − F)], where F, F_max_, and F_min_ are measured fluorescence intensities, and K_d_ is the dissociation constant of Fluo-3/AM, set as 400 nM.

#### Immunoblotting Analyses

Immunoblotting of target proteins by specific antibodies was used to quantify the changes in intracellular proteins phosphorylation which regulates various physiological functions (Korinek et al., 2021). Human neutrophils (2.5 × 10^7^ cells/ml) containing CaCl_2_ (1 mM) were incubated at 37°C, and FEN and CUMIN volatile oils (30 μg/ml) were added for 5 min, followed by the addition of fMLF for 25 s. The reaction was stopped by the addition of sample buffer (62.5 mM Tris–HCL, pH = 6.8, 2% SDS, 10% glycerol, 0.01% bromophenol blue, and 5% 2-mercaptoethanol). The cells were mixed and heated at 100°C for 15 min to fully lyse the cells and release the protein. The lysed cells were centrifuged at 14,000 rpm at 4°C for 20 min, and the supernatant was collected. Proteins were quantified and separated by SDS-PAGE gel electrophoresis using 12% acrylamide gel. The proteins were then transferred to a nitrocellulose membrane, which was soaked in 5% skim milk powder (in TBST containing 0.1% Tween 20) at room temperature for 1 hour to block nonspecific binding. Then, the membrane was immersed with corresponding primary antibodies in 5% BSA at 4°C overnight. Finally, the membrane was incubated with HRP secondary antibodies in 5% BSA at room temperature for 1 hour, and then enhanced chemiluminescence reagent (Millipore, Billerica, MA, United States) was added. The fluorescent bands of phosphorylated and total proteins were visualized using a densitometer (UVP Biospectrum Imaging System, LLC, Upland, CA, United States). The corresponding rabbit primary antibodies for p38, phospho-p38, JNK, phospho-JNK, Akt, phospho-Akt (Ser-473), ERK, phospho-ERK (1:2000), and HRP-linked anti-rabbit IgG antibodies (1:10000) were used for quantification of total and phosphorylated MAPK proteins. The quantitative ratios for all samples were normalized to the corresponding total protein.

### Antioxidant Assays

#### Superoxide Anion Scavenging Assay

The potential direct superoxide anion–scavenging capacity of the FEN and CUMIN samples was evaluated using a cell-free xanthine/xanthine oxidase system, as described before (Yang et al., 2017). Briefly, WST-1 (2-(4-iodophenyl)-3-(4-nitrophenyl)-5-(2,4-disulfophenyl)-2*H*-tetrazolium monosodium salt) at a concentration of 0.3 mM, tested samples, and 0.02 U/ml xanthine oxidase were added to the assay buffer containing 50 mM Tris (pH 7.4). Xanthine (0.1 mM) was added to the mixture at 30°C. A change in the absorbance was measured at 450 nm to detect the reduction level of the WST-1 superoxide anion.

#### Antioxidant ABTS and DPPH Assays

The antioxidant activity of FEN and CUMIN samples was determined utilizing ABTS and DPPH assays (Yang et al., 2017). The reaction was carried out in a mixture with the ethanol solution of ABTS (7 mM) or DPPH (100 μM) incubated with the tested volatile oils at 30°C. The absorbance was measured at 734 and 517 nm, respectively, and the effects were compared with the control.

### NRF2 Activity

Nuclear transcription factor NRF2 activity was evaluated in HacaT normal cells and Huh7 cancer cells according to a previously described method (Mykhailenko et al., 2020). The cell line HaCaT/ARE (antioxidant response element) was developed using a HaCaT cell line carrying a fragment derived from pGL4.37[luc2P/ARE/Hygro] plasmid and the luciferase reporter gene luc2P. The reporter cells were cultured in DMEM (Gibco BRL, Grand Island, NY, United States) supplemented with penicillin (100 U/ml), streptomycin (100 μg/ml), 10% heat-inactivated FBS (HyClone, Logan, UT, United States), and 100 μg/ml hygromycin at 37°C in 5% CO_2_. The cells were seeded (1 × 10^4^ cells/well) in a 96-well plate and treated with the sample for 18 h (single measurement). Resazurin (Cayman Chemical, Ann Arbor, MI, United States, final concentration of 0.1 mg/ml) was added, and the cells were incubated for an additional 4 h at 37°C. To determine cell viability, fluorescence of the reduced resazurin in the cells' supernatant (ex/em: 530/590 nm) was measured using a Synergy HT Multi-Mode Reader (BioTek, Winooski, VT, United States). The cells were then harvested, and the luciferase activity was measured according to the manufacturer's protocol (Promega Corporation, Madison, WI, United States). The luciferase activity was normalized to cell viability.

### Neuraminidase Activity Assay

A baculovirus containing neuraminidase NA9 on the surface (NA9-Bac) was used as a pseudotyped influenza virus model. An appropriate virus load of NA9-Bac was added into a 96-well plate and incubated with the extracts or compounds for 20 min at 37°C. Then, each well was supplemented with 25 μl of diluted fluorescent MUNANA substrate. After incubation for 30 min at ambient temperature, 150 μl of stop solution was added. The fluorescence intensity was detected immediately using Synergy HT Multi-Mode Microplate Reader (BioTek). Zanamivir, a known neuraminidase inhibitor, was used as a positive control.

### Lipid Droplet Assay

Lipid droplet assay was performed according to a previous method using a BSA-conjugated oleic acid system in Huh7 cells as described before (Yen et al., 2018). Briefly, cells seeded in μClear^®^ 96-well plates (Greiner Bio-ONE, Frickenhausen, Germany) were treated with oleic acid and the tested samples or DMSO for 18 h. Cells were stained with 2 μg/ml Hoechst 33342 and 1 μg/ml BODIPY^®^ 493/503 and fixed in paraformaldehyde. A High-content imaging (HCS) instrument was used to take and analyze images of the nuclei and lipid droplets (ImageXpress Micro System, Molecular Devices, Sunnyvale, CA, United States). The diameter settings were 8–25 μm for the nuclei and 0.5–2 μm for the lipid droplets.

### *In vitro* Assessment of the Antiviral Activity Against Enterovirus D68 and Influenza H1N1

Influenza H1N1 (A/WSN/33) virus–infected Madin-Darby canine kidney epithelial (MDCK) cell (Sethy et al., 2019) and enterovirus D68–infected rhabdomyosarcoma (RD) cell (Hsieh et al., 2020) experimental models were used to assess the cytopathic effects of the samples. Briefly, MDCK or RD cells (96-well plate, 2 × 10^4^ per well) were incubated in E10 medium at 5% CO_2_ for 16–24 h at 37°C and then washed once with Dulbecco’s phosphate-buffered saline before the infection step. The respective cells were infected with influenza or enterovirus at a ninefold median tissue culture infective dose, with or without the addition of the samples. The cells were treated with the volatile oil samples (50 µg/ml) for 72 h at 37°C. The cells were then fixed with 4% paraformaldehyde (PFA) for 1 h at room temperature and were stained using 0.1% crystal violet for 20 min. The optical density of the cells was measured with a VICTOR3™ microplate reader (PerkinElmer).

### *In vitro* Assessment of the Anti-allergic Activity

#### Chemicals and Reagents

DMEM high-glucose powder, [3-(4,5-dimethylthiazol-2-yl)-2,5-diphenyltetrazolium bromide], *p*-nitrophenyl-N-acetyl-D-glucosaminide (*p*-NAG), penicillin and streptomycin, dexamethasone, calcium ionophore A23187, mouse anti-DNP IgE antibody, and DMSO were purchased from Sigma-Aldrich (St. Louis, MO, United States). Moreover, FBS was obtained from HyClone (Logan, UT, United States). Dinitrophenyl-conjugated BSA (DNP-BSA) was purchased from Merck (Kenilworth, NJ, United States). All other chemicals and reagents were purchased at the highest purity and quality possible.

#### Cell Culture

The mucosal mast cell–derived rat basophilic leukemia (RBL-2H3) cell line was purchased from the American Type Culture Collection. The cells were grown in DMEM supplemented with 10% FBS and 100 U/ml penicillin plus 100 μg/ml streptomycin. The cells were cultured in 10-cm cell culture dishes (CELLSTAR) at 37°C in a humidified chamber with 5% CO_2_ in the air.

#### Cell Viability Assay

The methyl thiazole tetrazolium (MTT) assay (Marks et al., 1992) was used to measure the potentially toxic effects of the samples on RBL-2H3 cells according to a previous study (Korinek et al., 2016b). The degree of cell viability of each sample was calculated as the percentage of the control value (untreated cells). All experiments were repeated three times. The maximum tolerated dose of DMSO was 1%. Triton X-100 (0.5% solution) was used as the positive control, causing the death of all cells in a well.

#### Degranulation β-hexosaminidase Assay Induced by A23187 or an Antigen

The degree of A23187- and antigen-induced degranulation in RBL-2H3 cells was determined by a *β*-hexosaminidase activity assay as previously described (Chen et al., 2009; Korinek et al., 2016a; Korinek et al., 2017) with some modifications. Briefly, RBL-2H3 cells were seeded in a 96-well plate at a density of 2 × 10^4^ cells/well in a 96-well plate for the A23187 experiment or 3 × 10^4^ cells/well in a 48-well plate for the antigen-induced experiment. The cells were incubated at 37°C in 5% CO_2_ for at least 5 h to allow the attachment of the cells to the bottom of the wells. RBL-2H3 cells for the antigen-induced assay were sensitized with anti-DNP IgE (0.1 μg/ml) overnight. The cells were then treated by various concentrations of the sample or 1% DMSO in 100 μl medium (untreated control), followed by 20 h of incubation at 37°C in 5% CO_2_. The cells were washed twice by pre-warmed Tyrode's buffer (135 mM NaCl, 5 mM KCl, 1.8 mM CaCl_2_, 1.0 mM MgCl_2_, 5.6 mM glucose, 20 mM HEPES, and 1 mg/ml BSA at pH 7.4) and stimulated by calcium ionophore A23187 (1 μM) or the cross-linking antigen DNP-BSA (100 ng/ml) diluted in Tyrode's buffer. The cells were incubated at 37°C in 5% CO_2_ for 1 h. Unstimulated cells were lysed with 0.5% Triton X-100 solution to achieve the total release of *β*-hexosaminidase. Untreated unstimulated cells served as a spontaneous source for the release of *β*-hexosaminidase. Dexamethasone (10 nM) was used as a positive control. To detect the amount of the released *β*-hexosaminidase, aliquots of the supernatants of the cells (50 μl) were collected and were incubated with an equal volume (50 μl) of the substrate, *p*-NAG (1 μM) prepared in citrate buffer (0.1 M, pH 4.5). After 1 h incubation at 37°C, the reaction was stopped by the addition of 100 μl of stop buffer (0.1 M Na_2_/NaHCO_3_, pH 10.0). The absorbance was measured at 405 nm on a microplate reader (Thermo Multiscan Reader). The inhibition percentage of *β*-hexosaminidase release from RBL-2H3 cells was calculated as the percentage of the control value (untreated stimulated cells) using the following equation:

Inhibition (%) = [1 − (ODsample − ODspontaneous)/(ODcontrol − ODspontaneous)] × 100.

### GC-MS Analyses

Volatile oil samples were analyzed by the GC-MS electron ionization method using column GsBP-5MS. Helium was used as a carrier gas at a flow rate of 1.0 ml/min in split mode (1:20). The initial column temperature of 75°C was maintained for 5 min and then ramped up at a heating rate of 15°C/min to reach 300°C and held for 20 min. The mass spectrometer was operated in the EI mode at 70 eV with a mass scanning range of 50–500 and source temperature of 230°C. The standard hydrocarbon mixture of C8–C40 *n*-alkanes (Multi-State Hydrocarbon Window Defining Standard, AccuStandard, AS-DRH-008S-R1) was used to set the reference points for calculations of the Kovats retention indexes (KIs) derived from a non-polar (Rtx-5MS) capillary column. The identity of each compound was determined by comparing its KIs based on *n*-alkanes hydrocarbon standard and the NIST library database (NIST). The quantitative analysis expressing the percentage of the identified components in each volatile oil was obtained by the integration of the peak areas using Thermo software. Only fully identified compounds are reported in this study.

### Statistical Analyses

Data are presented as means ± SEM of at least three independent measurements unless otherwise indicated. Comparisons were carried out using Student's *t*-test. The 50% inhibitory concentration (IC_50_) was calculated from the dose–response curve obtained by plotting the percentage of inhibition versus concentration (linear function, Microsoft Office). All statistical analyses were done, and the graphs were plotted using SigmaPlot software (Jandel Scientific, San Rafael, CA) or GraphPad Prism software. Values with *p < 0.05, **p < 0.01, ***p < 0.001 were considered statistically significant.

## Results

### Screening Various Bioactivities

The volatile oil samples were screened for a plethora of bioactivities, including NFR2 expression in normal and cancer cells, virus-related neuraminidase activity, lipid droplets accumulation, anti-allergic degranulation assays in mast cells, and the protective effects against influenza H1N1 and enterovirus D68. Cumin volatile oil exhibited a 36% inhibitory effect on the lipid droplets accumulation in Huh7 cells. Other results showed only minor effects at the indicated concentrations (Table 1). The obtained results suggested that higher concentrations should be explored in any future experiments.

**TABLE 1:** Bioactivity screening of volatile oils common in the Mediterranean diet.

Sample	Description	Relative NRF2 activity^a^ in HacaT cells^b^	Relative NRF2 activity^a^ in Huh7 cells^c^	Neuraminidase inhibition activity^d^	Lipid droplet inhibition activity^e^	A23187-induced degranulation assay in RBL-2H3 cells^f^	Antigen-induced degranulation assay in RBL-2H3 cells^f^	Protective activity against Influenza H1N1, MDCK cells^g^	Protective activity against Enterovirus D68, RD cells^g^
**FEN**	fennel vol. oil	90.3	72.0	−1.5 ± 5.0	79.3 ± 25.1	NA	NA	NA	NA
**CUMIN**	cumin vol. oil	112.6	92.6	−5.3 ± 2.8	**64.4 ± 18**	NA	NA	NA	NA
**MAJO**	marjoram vol. oil	68.8	72.4	−6.8 ± 3.3	82.8 ± 13.3	NA	NA	NA	NA
**CAR**	caraway vol. oil	96.2	101.7	−3.4 ± 4.6	76.9 ± 15.9	NA	NA	NA	NA
**ANIS**	anise vol. oil	76.3	73.7	−6.7 ± 1.8	81.7 ± 21.5	NA	NA	NA	NA
**LAV**	lavender vol. oil	91.1	78.9	−1.7 ± 7.0	86.6 ± 26.8	NA	NA	NA	NA
**TBHQ** ^h^	NRF2 positive control	684.3 ± 53.3	—	—	—	—		—	—
**Luteolin** ^i^	NRF2 negative control	—	23.8 ± 0.3	—	—	—		—	—
**Zanamivir** ^j^	neuraminidase positive control	—	—	97.4 ± 0.0	—	—		—	—
**TC** ^k^	lipid droplet positive control	—	—	—	16.3 ± 0.2	—		—	—

a Relative luciferase activity was calculated by normalizing luciferase activity to cell viability and presented as the fold to solvent control. (%, mean ± SD, *n* = 1).

b HacaT, a normal skin cell line. The volatile oil concentration was 100 µg/ml.

c Huh7, a liver cancer cell line. The volatile oil concentration was 100 µg/ml.

d Neuraminidase inhibition assay (%, mean ± SD, *n* = 2). The volatile oil concentration was 100 µg/ml.

e Lipid droplet count (%, mean ± SD, *n* = 1). The average lipid droplet counts/cells of oleic acid were used as standard representing 100% of lipid loading in the Huh7 cell line. The volatile oil concentration was 100 µg/ml.

f Anti-allergic assay was evaluated based on beta-hexosaminidase release from the mast cells induced by A23187 or antigen (%, mean ± SD, *n* = 3). The volatile oil concentration was 100 µg/ml. Dexamethasone (10 nM), a positive control, inhibited 70.3% of A23187-and 69.2% of antigen-induced β-hexosaminidase release. NA, not active (< 20% inhibition).

g The protective effects were evaluated based on the viability of cells infected by the virus. The volatile oil concentration was 50 µg/ml. NA, not active.

h TBHQ, 2-(1,1-dimethylethyl)-1,4-benzenediol, was used as the positive control for Nrf2 activation. The drug concentration was 10 µM.

i Luteolin was used as a negative control for Nrf2 activation. The drug concentration was 50 µM.

j Zanamivir was used as a positive control for neuraminidase inhibition. The drug concentration was 1 µM.

k TC, Triacsin C, an inhibitor of long fatty acyl CoA synthetase, was used as a positive control for lipid droplet inhibition. The drug concentration was 1 µM.

FEN, volatile oil from fennel (*Foeniculum vulgare*); CUMIN, volatile oil from cumin (*Cuminum cyminum*); MAJO, volatile oil from marjoram (*Origanum majorana*); CAR, volatile oil from caraway (*Carum carvi*); ANIS, volatile oil from anise (*Pimpinella anisum*); LAV, volatile oil from lavender (*Lavandula angustifolia*). Bold value represents active volatile oil sample.

#### Anti-allergic Assay

The volatile oil samples showed no toxicity (up to 100 μg/ml) toward RBL-2H3 (rat basophilic leukemia cells) cells using the MTT viability assay. The anti-allergic assay depends on the release of *β*-hexosaminidase from the granules in RBL-2H3 cells induced by calcium ionophore (A23187) or antigen (anti-DNP IgE plus DNP-BSA). Our results indicated no significant effect in both A23187-induced and antigen-induced degranulation assays at 100 μg/ml (Table 1).

#### Protective Effects Against Influenza H1N1 and Enterovirus D68

Influenza H1N1 (A/WSN/33) virus-infected MDCK epithelial cells and RD cell models were used to evaluate the volatile oils protective effects against influenza H1N1 or enterovirus D68 infection, respectively. None of the volatile oils (50 µg/ml) showed any protective effects against both viruses (Table 1).

### Antimicrobial Activity

The antimicrobial activity of the volatile oils was assessed by measuring the inhibition zone of pathogenic strains (*S*. *aureus* ATCC 12600, *E. coli* ATCC 11775, *C. albicans* ATCC 90028, *V. harveyi* ATCC 25919, *P. aeruginosa* ATCC 31156, and *B. subtilis* ATCC 6633) in Petri dishes using agar-block assay and MIC assay. The values were then calculated to indicate MIC of the volatile oils against the pathogenic strains (Table 2). Several pathogenic strains including gram-negative *E. coli* and *V. harveyi*, and gram-positive *B. subtilis* were susceptible to the volatile oils treatment of CUMIN, MAJO, and ANIS with MIC values ranging from 128 to 512 μg/ml. The results indicated that MAJO exhibited comparable activity against *V. harveyi* to the positive control, streptomycin. These data indicate promising adjuvant effects of these volatile oils in the treatment of infections caused by bacteria. However, the indicated concentrations of the volatile oils did not inhibit the growth of the gram-positive *S. aureus* and *C. albicans* (yeast), or the gram-negative *P. aeruginosa*.

**TABLE 2:** Antimicrobial effects of volatile oils on six different bacterial strains.

Sample	Description	MIC (μg/ml)					
		*S. aureus*	*E. coli*	*C. albicans*	*V. harveyi*	*P. aeruginosa*	*B. subtilis*
**FEN**	fennel volatile oil	NA	NA	NA	NA	NA	NA
**CUMIN**	cumin volatile oil	NA	**512**	NA	**512**	NA	**256**
**MAJO**	marjoram volatile oil	NA	**256**	NA	**256**	NA	**128**
**CAR**	caraway volatile oil	NA	NA	NA	NA	NA	NA
**ANIS**	anise volatile oil	NA	**512**	NA	**512**	NA	**512**
**LAV**	lavender volatile oil	NA	NA	NA	NA	NA	**512**
**streptomycin**	positive control	8	16	NA	256	32	16
**penicillin G**	positive control	8	0.0625	8	8	NA	0.0625

Results are presented as minimum inhibitory concentration (MIC) values from three independent experiments. NA, not active at 512 μg/ml.

FEN, volatile oil from fennel (*Foeniculum vulgare*); CUMIN, volatile oil from cumin (*Cuminum cyminum*); MAJO, volatile oil from marjoram (*Origanum majorana*); CAR, volatile oil from caraway (*Carum carvi*); ANIS, volatile oil from anise (*Pimpinella anisum*); LAV, volatile oil from lavender (*Lavandula angustifolia*). Bold values represent active volatile oil samples.

### Anti-inflammatory Activity

#### Respiratory Burst and Degranulation Assays

First, the anti-inflammatory assays were performed utilizing fMLF/CB-induced superoxide anion generation and elastase release by human neutrophils (Table 3). Among all tested volatile oils, fennel (FEN) and cumin (CUMIN) volatile oils exerted potent anti-inflammatory activity. FEN exerted the highest inhibitory effect on fMLF/CB-induced superoxide anion generation with an IC_50_ of 3.81 μg/ml, while CUMIN showed an IC_50_ of 16.67 μg/ml. FEN at 10 μg/ml suppressed superoxide anion generation by 86.3% (Table 3). In the elastase release assay, both FEN and CUMIN showed significant effects with similar potency, IC_50_ of 17.16 and 16.24 μg/ml, respectively. FEN demonstrated a more potent effect on respiratory burst (IC_50_ of 3.81 μg/ml) than the degranulation assay (IC_50_ of 17.16 μg/ml), showing a similar pattern of inhibition as genistein, a known tyrosine kinase inhibitor, which was used as a positive control in the superoxide and elastase release assays (IC_50_ values 0.47 and 12.05 μg/ml, respectively). Among other volatile oils, caraway significantly inhibited elastase release by 38.60 ± 2.49% at 30 μg/ml.

**TABLE 3:** Effects of volatile oils on superoxide anion generation and elastase release in FMLP/CB-induced human neutrophils.

Sample	Description	Superoxide anion	Elastase release
		IC_50_ (μg/ml)^a^	IC_50_ (μg/ml)^a^
**FEN**	fennel volatile oil	**3.81** ± **0.34** ^b^	**17.16** ± **3.95**
**CUMIN**	cumin volatile oil	**16.67** ± **4.10**	**16.24** ± **1.13**
**MAJO**	marjoram volatile oil	>10	>10
**CAR**	caraway volatile oil	>10	>10^c^
**ANIS**	anise volatile oil	>10	>10
**LAV**	lavender volatile oil	>10	>10
**genistein**	positive control	0.47 ± 0.14	12.05 ± 1.64

a Concentration necessary for 50% inhibition (IC_50_). The values were calculated based on testing various concentrations of samples in three independent experiments.

b FEN inhibited superoxide anion generation by 86.33 ± 2.33% at 10 μg/ml.

c CAR significantly inhibited elastase release by 38.60 ± 2.49% at 30 μg/ml.

FEN, volatile oil from fennel (*Foeniculum vulgare*); CUMIN, volatile oil from cumin (*Cuminum cyminum*); MAJO, volatile oil from marjoram (*Origanum majorana*); CAR, volatile oil from caraway (*Carum carvi*); ANIS, volatile oil from anise (*Pimpinella anisum*); LAV, volatile oil from lavender (*Lavandula angustifolia*). Bold values represent active volatile oil samples.

**FIGURE 1:**
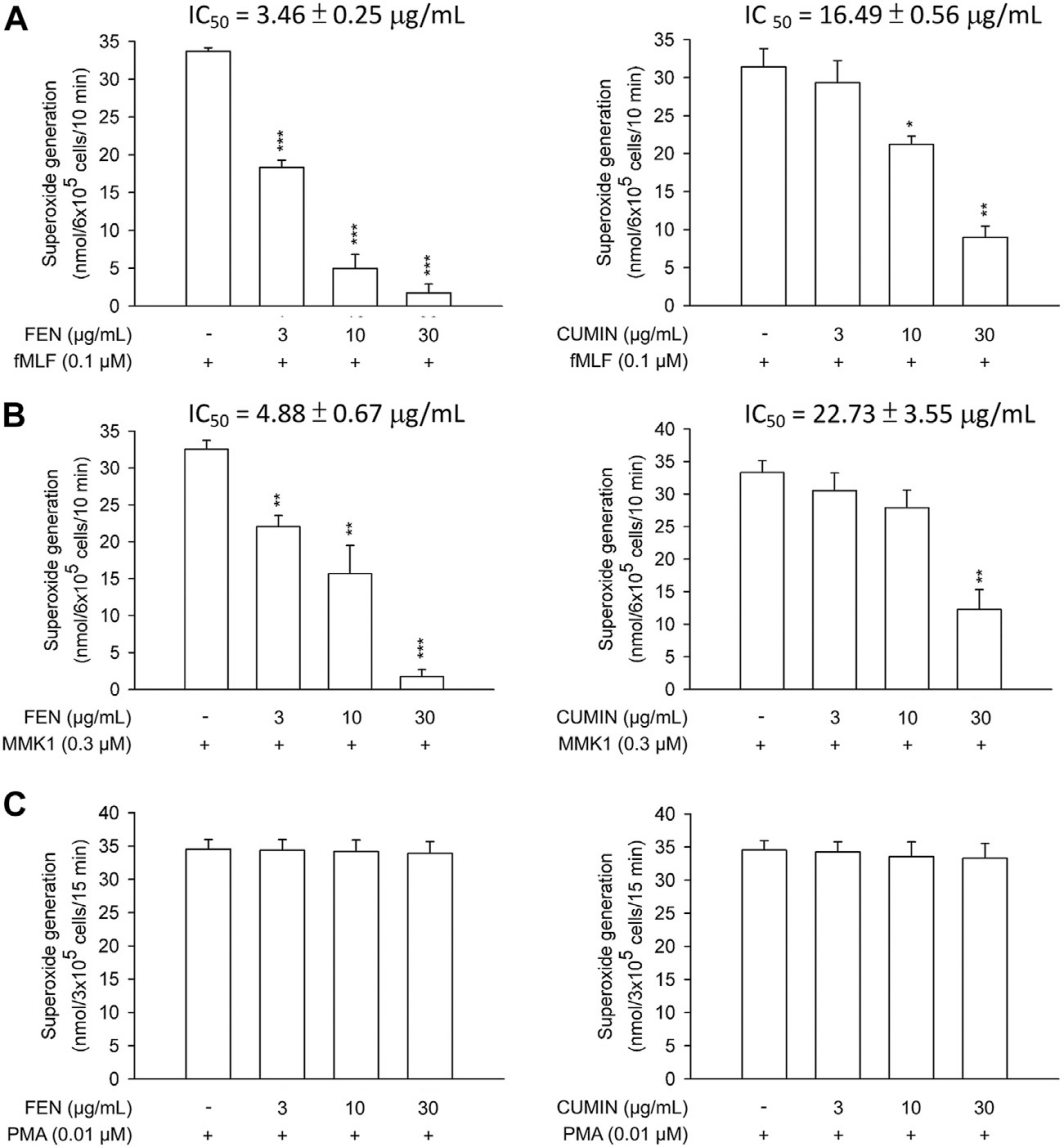
Effects of fennel volatile oil (FEN) (left panel) and cumin volatile oil (CUMIN) (right panel) on respiratory burst in human neutrophils using various inducers. Human neutrophils were incubated with 0.1% DMSO (as control), fennel or cumin volatile oils for 5 min, and then activated by **(A)** fMLF, **(B)** MMK1, or **(C)** PMA in the presence of CB (except PMA) for another 10 min (details in *Measurement of Superoxide Generation*). Superoxide anion generation was detected by cytochrome *c* reduction spectrophotometrically at 550 nm. Data are expressed as mean ± S.E.M. (*n* = 3). *p < 0.05; **p < 0.01; ***p < 0.001 compared with the control (inducer only).

**FIGURE 2:**
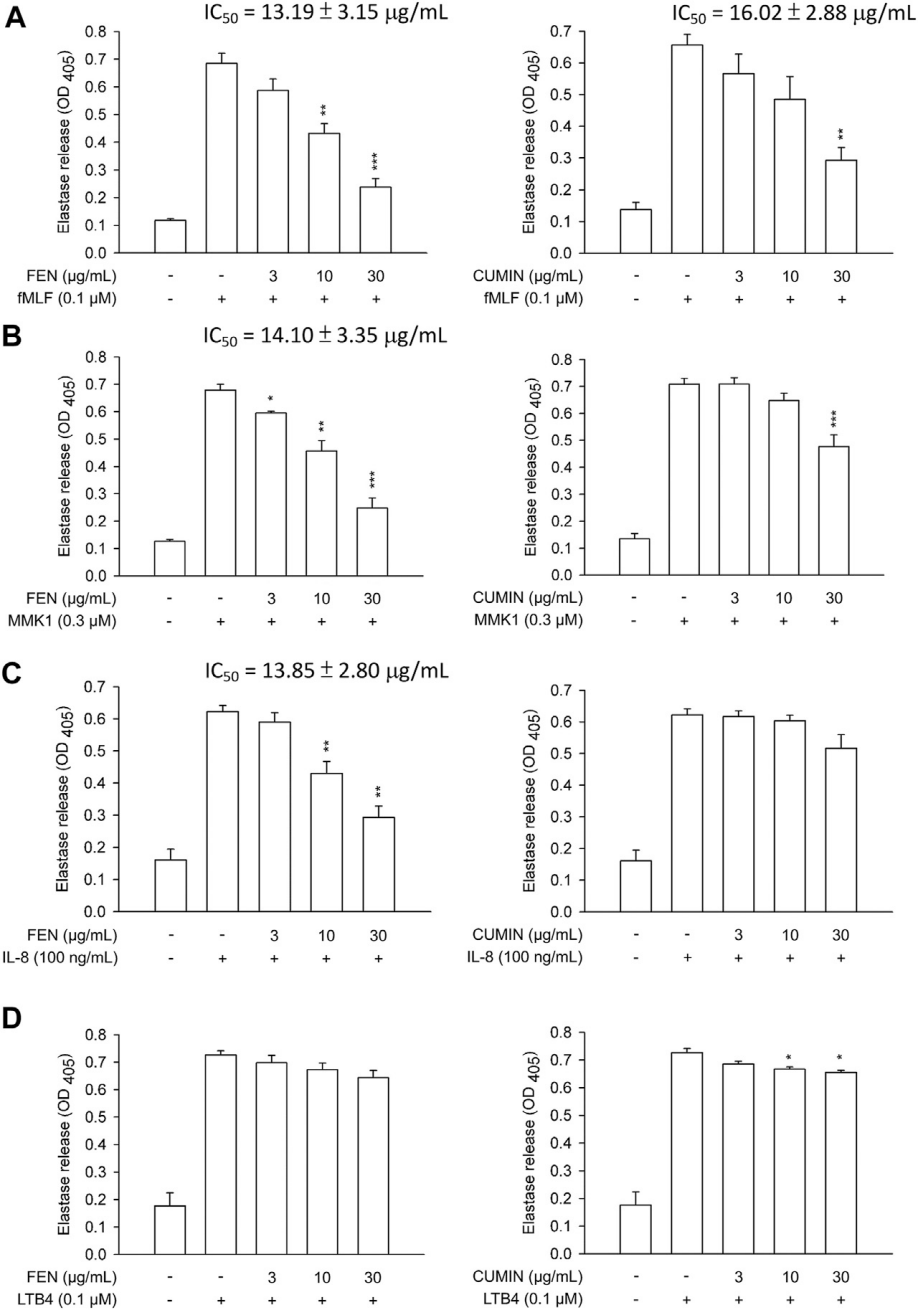
Effects of fennel (FEN) (left panel) and cumin (CUMIN) (right panel) volatile oils on degranulation in human neutrophils using various inducers. Human neutrophils were incubated with 0.1% DMSO (as control), fennel or cumin volatile oils for 5 min, and then activated by **(A)** fMLF, **(B)** MMK1, **(C)** IL-8, or **(D)** LTB4 in the presence of CB for another 10 min (details in *Measurement of Elastase Release*). Elastase release was measured based on consumption of the substrate present in the medium and OD was read at 405 nm. Data are expressed as mean ± S.E.M. (*n* = 3). *p < 0.05; **p < 0.01; ***p < 0.001 compared with the control (inducer only).

Next, the effects of FEN and CUMIN volatile oils were examined on the addition of various inducers serving as neutrophil chemoattractants that act via different mechanisms using the same superoxide anion generation (Figure 1) and elastase release (Figure 2) assays. The formyl peptide receptor (FPR) plays an important role in inflammatory signal transduction and neutrophil activation (Yang and Hwang, 2016). Formyl peptides have been found in pathogenic bacteria as well as in the human body as endogenous mitochondrial proteins (Carp, 1982). In this experiment, both FEN and CUMIN were confirmed to have a dose-dependent inhibitory effect on both superoxide and elastase release induced by fMLF, an FPR-1 agonist (Figure 1A and Figure 2A, IC_50_ range 3.5–16.5 μg/ml). When MMK1, an FPR-2 agonist, was used to activate neutrophils, both FEN and CUMIN also significantly suppressed superoxide generation and elastase release (Figure 1B and Figure 2B, IC_50_ range 4.9–22.7 μg/ml). In MMK1-induced superoxide anion generation assay, FEN showed the most potent inhibition (IC_50_ 4.9 μg/ml), while the effect of CUMIN was much weaker (IC_50_ 22.7 μg/ml) (Figure 1B). In sum, similar inhibitory potencies of FEN and CUMIN on both FPR-1 and FPR-2 agonists–activated neutrophils indicated a promising anti-inflammatory potential, acting partly via the inhibition of G-protein receptors FPR. However, FEN exhibited markedly stronger effects on FPR inducers–mediated superoxide anion generation in comparison with CUMIN. Interestingly, FEN also exerted potent inhibitory activity in IL-8–induced elastase release assay with IC_50_ 13.9 μg/ml, while CUMIN was inactive (Figure 2C). IL-8 acts as a neutrophil chemoattractant binding to CXCR1, a ligand-bound chemokine receptor (Lacy, 2006). The results suggest the potential effect of FEN in inhibiting CXCR1, unlike CUMIN. No effect was observed on PMA-induced superoxide generation (Figure 1C), suggesting that FEN and CUMIN volatile oils did not suppress the PKC pathway. The insignificant effect of FEN and CUMIN on elastase release stimulated by LTB-4, a BLT1 receptor agonist, suggests different molecular targets (Figure 2D).

**FIGURE 3:**
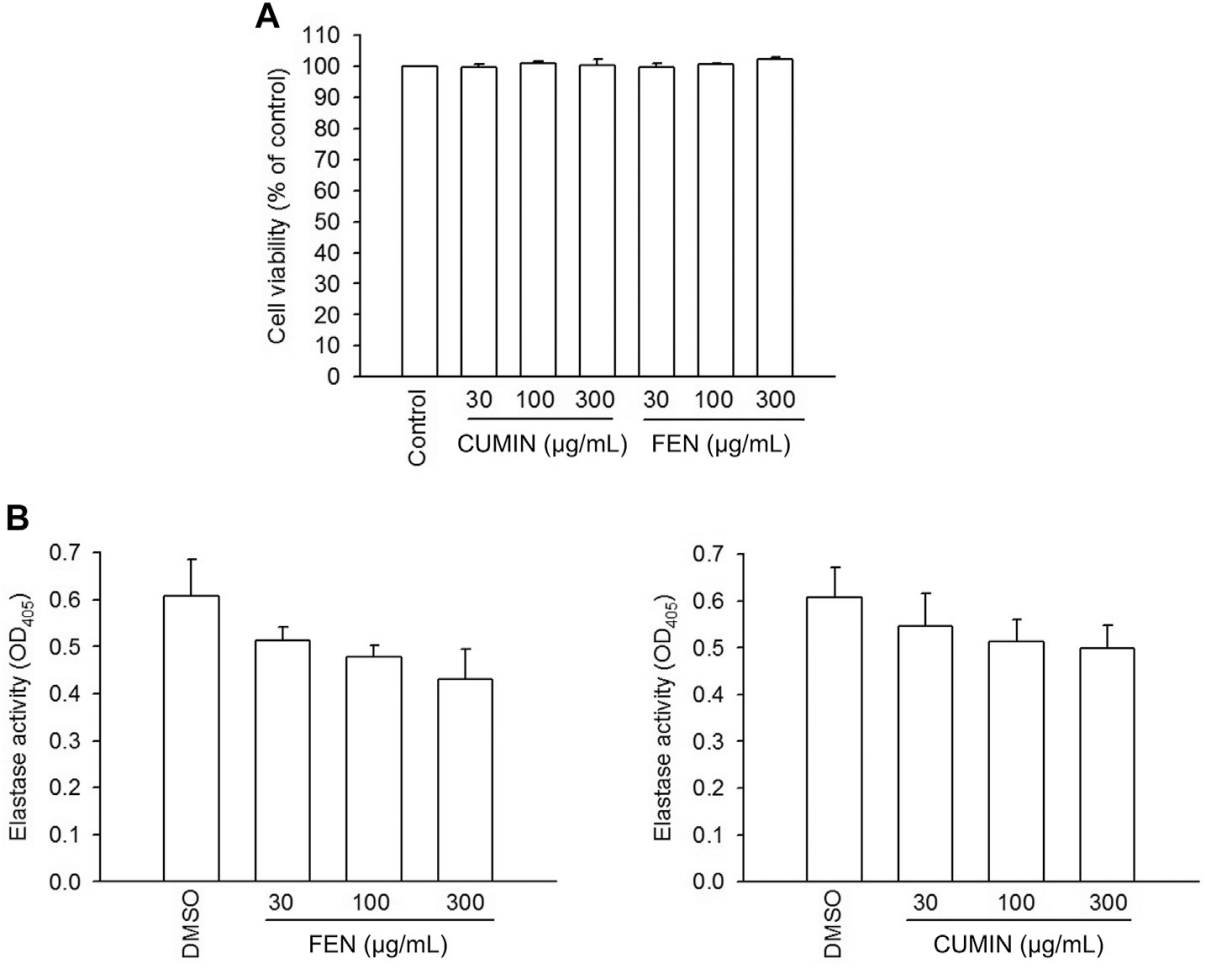
LDH release viability assay in human neutrophils, and elastase enzymatic activity in cell-free system. **(A)** Human neutrophils were incubated with 0.1% DMSO (as control), fennel (FEN, 30–300 μg/ml), or cumin (CUMIN, 30–300 μg/ml) volatile oils, for 15 min. The evaluation of cell viability was based on LDH release, which was expressed as a percentage of enzyme released by samples treatment compared with untreated control (DMSO) and a total release (0.1% Triton X-100). The analysis was performed by using an enzyme-associated immunosorbent assay and OD was read at 490 nm. The data are shown as mean ± S.E.M. (*n* = 3). **(B)** Human neutrophils were incubated with fMLF/CB for 15 min. The elastase supernatant was obtained and incubated with DMSO (as control), fennel (FEN, 30–300 μg/ml), or cumin (CUMIN, 30–300 μg/ml) volatile oils for 2 min before the addition of substrate (100 μM). Elastase activity was measured at 405 nm. Data are presented as means ± S.E.M. (*n* = 3). *p < 0.05 compared with the control group.

Furthermore, to understand whether FEN and CUMIN volatile oils produced toxic effects on human neutrophils, an LDH release assay was employed, and none of the volatile oils (30–300 μg/ml) affected the viability of the cells (Figure 3A). Therefore, the toxic effect of the tested volatile oils could be excluded.

The fact that both FEN and CUMIN were either active or inactive when the cells were stimulated by various inducers to trigger elastase release suggests no direct effect on elastase enzymatic activity, which was confirmed using elastase cell-free assay (Figure 3B, 30–300 μg/ml). This indicates that FEN and CUMIN are capable of inhibiting degranulation process in human neutrophils.

#### Scavenging Effects

**FIGURE 4:**
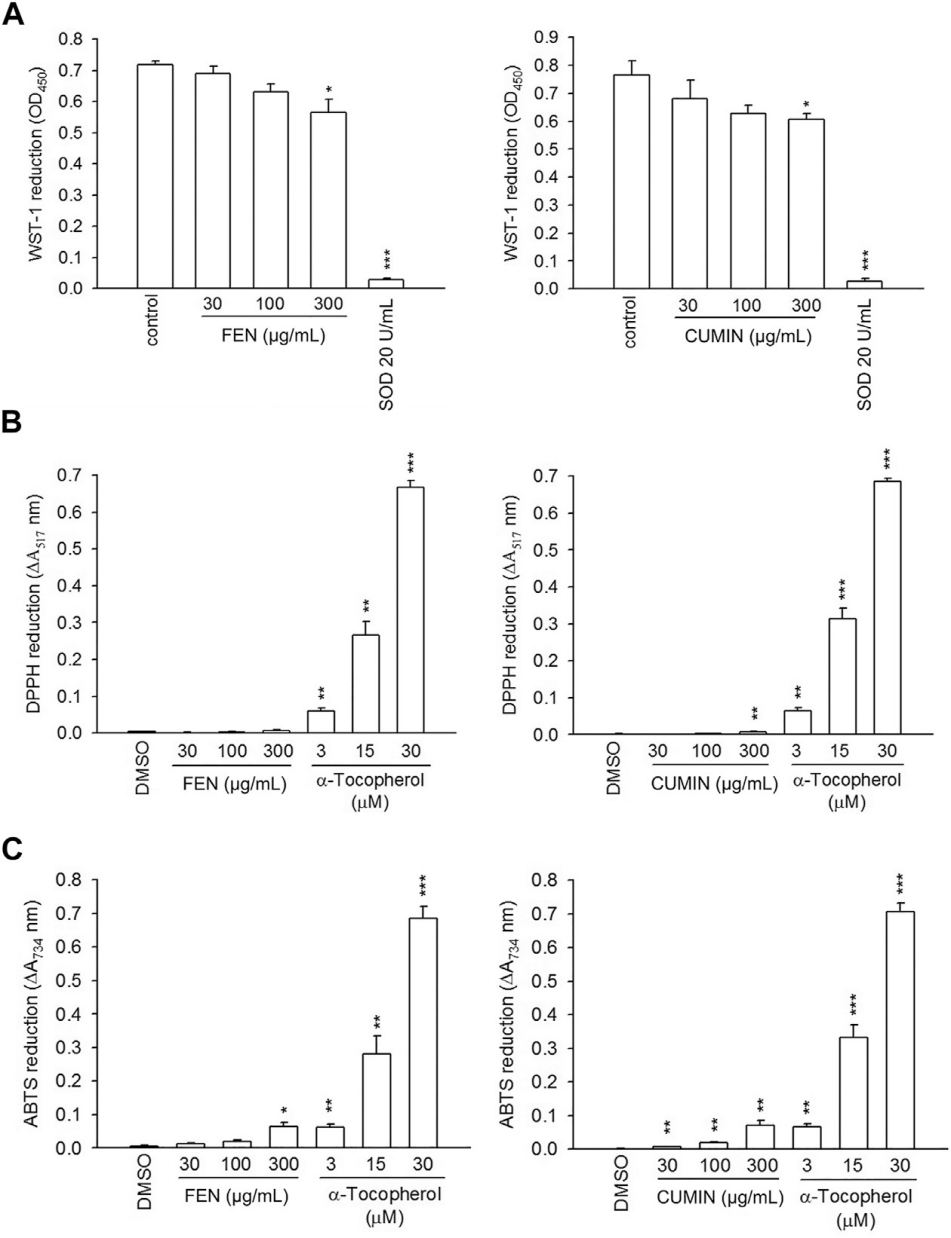
The effects of fennel (FEN) (left panel) and cumin (CUMIN) (right panel) volatile oils on xanthine oxide and antioxidant assays in a cell-free system. Reduction of **(A)** WST-1, **(B)** DPPH, and **(C)** ABTS was measured at 450 nm, 517 nm, and 734 nm, respectively. SOD (WST-1) and α-tocopherol (DPPH and ABTS) were used as positive controls. Data are expressed as mean ± S.E.M. (*n* = 3). *p < 0.05; **p < 0.01; ***p < 0.001 compared with the control (DMSO).

Volatile oils are often appraised for their many biological effects that may be correlated to their potent antioxidant potential (Bozin et al., 2006; Rather et al., 2016). To evaluate the volatile oils' antioxidant activities, their scavenging effects on the superoxide anion was determined using xanthine/xanthine oxidase cell-free assay (Figure 4A). No effect was detected for both FEN and CUMIN (30–300 μg/ml) compared with SOD (positive control). We further evaluated the antioxidant capacity of FEN and CUMIN in reducing DPPH (Figure 4B) and ABTS (Figure 4C) radicals. Both volatile oils (30–300 μg/ml) demonstrated no (DPPH) to very weak (ABTS) antioxidant activity when compared with *α*-tocopherol, the positive control.

These results indicate that FEN and CUMIN exhibited an inhibitory effect on respiratory burst and degranulation function of neutrophils but did not exert superoxide- and radical-scavenging or ligand–enzyme interaction effects.

#### Effects of FEN and CUMIN on PKA Pathway

**FIGURE 5:**
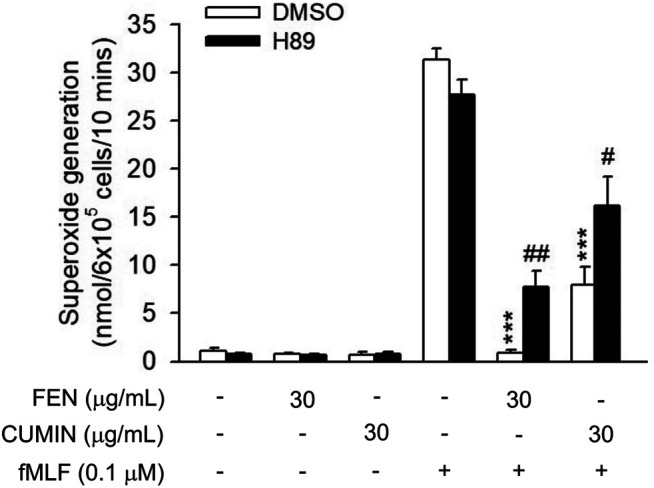
Effects of PKA inhibitor H89 on FEN- and CUMIN-caused inhibition of superoxide generation in human neutrophils. H89 (3 μM) were preincubated for 5 min before the addition of fennel (FEN) or cumin (CUMIN) volatile oils (30 μg/ml). Superoxide generation was induced by FMLP/CB and measured using SOD-inhibitable cytochrome *c* reduction (details in *Measurement of Superoxide Generation*). ***p < 0.001 compared with the control group (fMLF/CB control). #*p* < 0.05, ##*p* < 0.05 compared with the control group (CUMIN- or FEN-inhibited superoxide generation).

It is known that the cAMP pathway has a negative feedback effect on neutrophils activation (Hwang et al., 2006b). To examine whether cAMP is involved in the inhibitory effect of FEN and CUMIN, the protein kinase (PKA) inhibitor H89 was used. Interestingly, the PKA inhibitor H89 partially restored the superoxide anion generation inhibited by both FEN and CUMIN (30 μg/ml, Figure 5), suggesting cAMP and PKA were at least partly involved in the anti-inflammatory effects of CUMIN and FEN.

#### Effect of FEN and CUMIN on fMLF-Stimulated Calcium Modulation in Human Neutrophils

**FIGURE 6:**
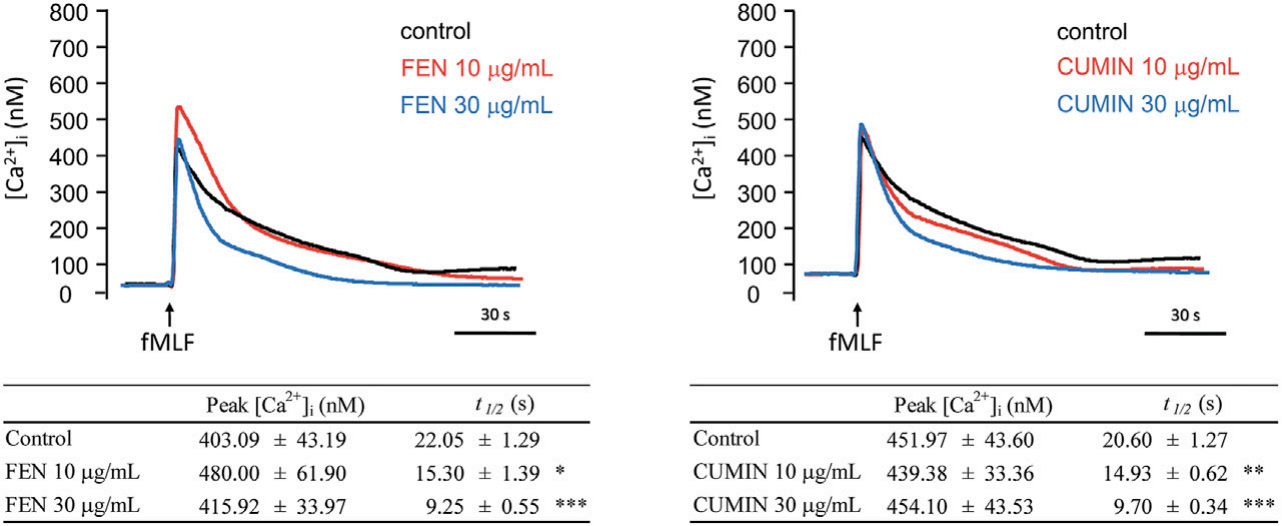
Fennel (FEN) (left) and cumin (CUMIN) (right) volatile oils inhibit calcium mobilization in fMLF-activated human neutrophils. A Fluo-3/AM-labeled neutrophils were incubated with DMSO (as control), fennel (FEN, 10–30 μg/ml, left), or cumin (CUMIN, 10–30 μg/ml, right) volatile oils and then activated with fMLF (0.1 μM) in the presence of Ca^2+^ (1 mM). Fluorescence was continuously monitored at 37°C with stirring. All data are presented as mean ± S.E.M. (*n* = 4–6). *p < 0.05, *p < 0.01, ***p < 0.001 compared with the control (fMLF only).

When neutrophilic G-protein receptor FPR is stimulated, a downstream molecular cascade is activated via calcium mobilization (inositol-1,4,5-triphosphate (IP3) and PLC pathways), followed by the release of effector molecules. The effect of FEN and CUMIN (10–30 μg/ml) on intracellular calcium concentration ([Ca^2+^]_i_) in neutrophils stimulated by fMLF was evaluated. Under physiological conditions in the presence of extracellular calcium, the activation would cause a transient increase in [Ca^2+^]_i_ until reaching a maximum ([Ca^2+^]_i_ peak) which subsequently returns [Ca^2+^]_i_ to equilibrium, usually within a minute. Experimental results illustrate that FEN and CUMIN significantly shortened the calcium recovery time t_1/2_, indicating a downstream effect on neutrophils activation (Figure 6).

#### Effect of FEN and CUMIN on MAPKs Downstream Signaling Pathways

**FIGURE 7:**
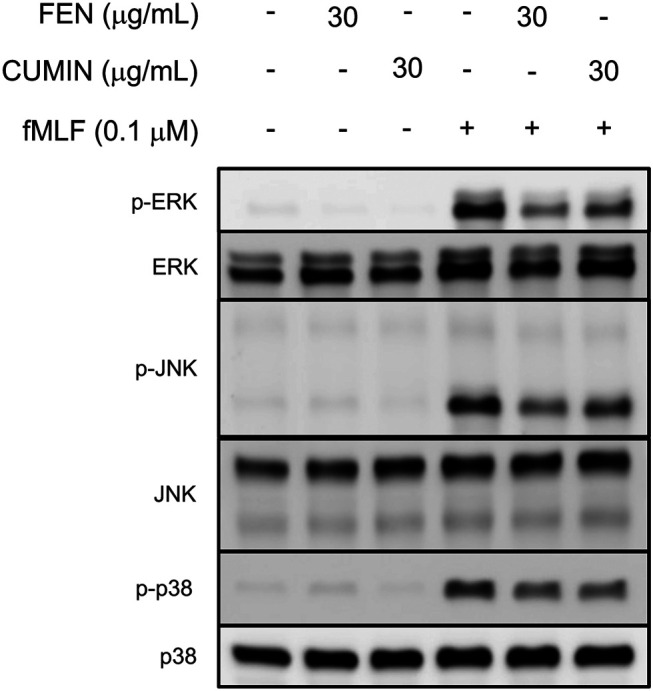
Fennel (FEN) and cumin (CUMIN) volatile oils inhibit fMLF-induced phosphorylation of p38, ERK, or JNK in human neutrophils. Human neutrophils were incubated with DMSO (as control), fennel (FEN, 30 μg/ml), or cumin (CUMIN, 30 μg/ml) volatile oils for 5 min and stimulated with or without fMLF for 25 s. Phosphorylation of ERK, JNK, and p38 was analyzed by immunoblotting with antibodies raised against the phosphorylated and total form of each protein (*n* = 4).

The activation and phosphorylation of signaling proteins, such as MAPK, regulate physiological functions of neutrophils, including respiratory burst, degranulation, or adhesion. Immunoblotting experiments revealed that FEN and CUMIN (30 μg/ml) significantly inhibited the phosphorylation of MAPK (p38, ERK, JNK) proteins in fMLF-activated human neutrophils (Figure 7). This result demonstrates the inhibitory effects of CUMIN and FEN on FPR downstream signaling.

### GC-MS Analysis of Volatile Oils

**FIGURE 8:**
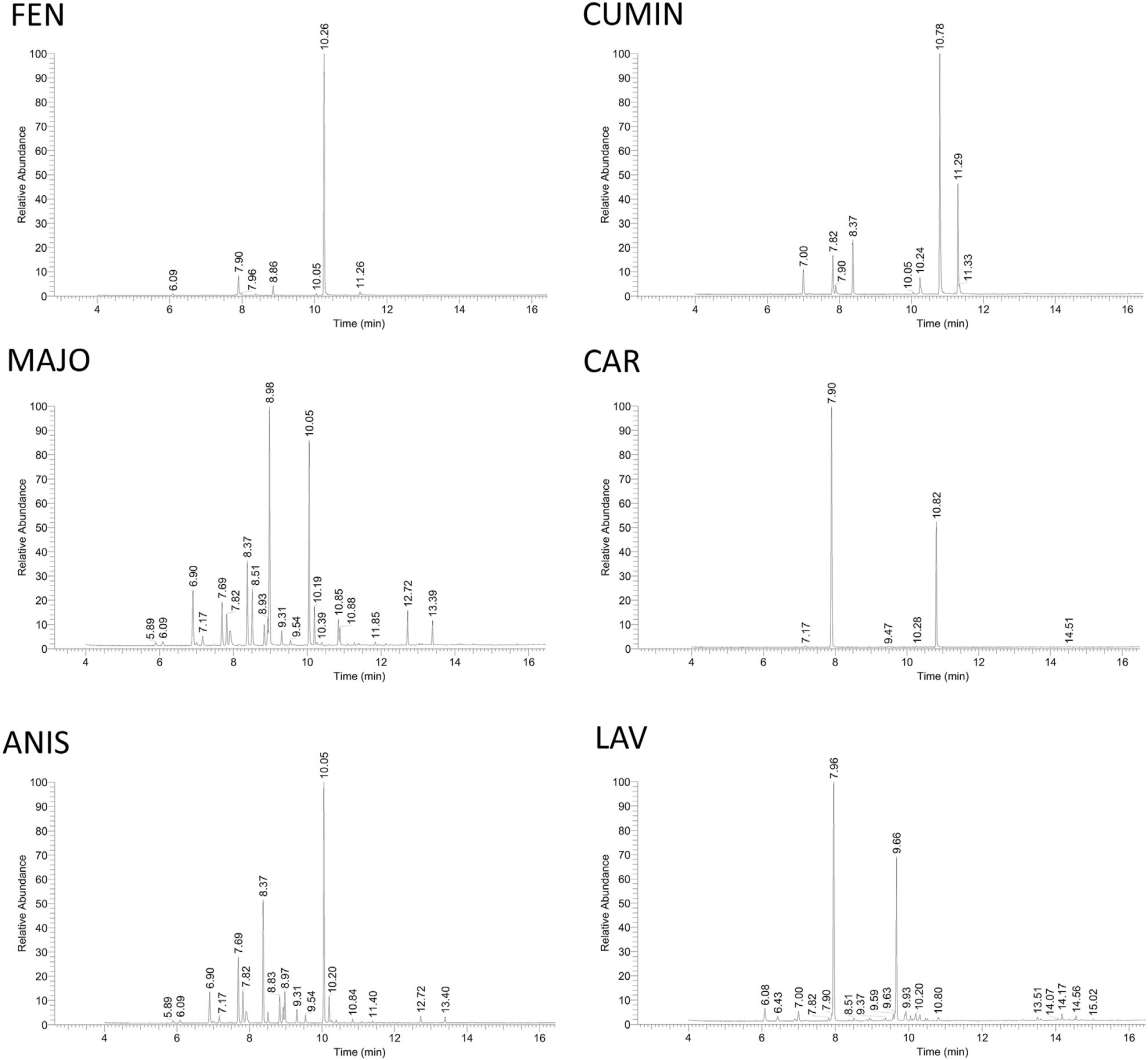
GC-MS analysis of volatile oils. The quantitative analysis was done by integrating peaks of each detected compound using Thermo Scientific Excalibur software. The identity of compounds was assessed by comparison of their mass data with NIST library data, literature as well as calculated Kovat's retention index based on standard hydrocarbon mixture (see Table 4). FEN, fennel volatile oil; CUMIN, cumin volatile oil; MAJO, marjoram volatile oil; CAR, caraway volatile oil; ANIS, anise volatile oil; LAV, lavender volatile oil.

Furthermore, the volatile content of the fennel, cumin, marjoram, caraway, anise, and lavender volatile oils was evaluated by GC-MS analysis, and the results are illustrated in Figure 8 and Table 4. The compounds identification was based on comparison of mass data and calculated retention indices with literature (Table 4 and Supplementary Figures S1–S6). Among the most active volatile oils, the GC-MS analysis of fennel volatile oil (FEN) revealed the presence of estragole (84.5%) and limonene (7.0%), while only 1.9% was accounted for anethole (Supplementary Figure S1). Interestingly, anethole is usually a predominant component of fennel volatile oils representing 50–85% of the content among various compounds reported in fennel seeds from different sources (Cosge et al., 2008; Badgujar et al., 2014; Murbach Teles Andrade et al., 2014; Ma et al., 2015). However, there is a major difference between diverse fennel samples from different geographical sources. Our results bode well with a previous report that identified a large amount of estragole in volatile oil obtained from Egyptian fennel, while the content of anethole was minor (Afifi et al., 2021). Importantly, estragole was reported as a potential genotoxic carcinogen (Afifi et al., 2021), and its high content in FEN must be taken into consideration for any future therapeutic use.

**TABLE 4:** GC-MS analysis of volatile oils.

Retention time (min)	Name	Molecular weight	Formula	Kovat's index exp.	Kovat's index ref.	FEN %	CUMIN %	MAJO %	CAR %	ANIS %	LAV %
6.09	*α*-pinene	136	C_10_H_16_	948	943	0.67	0.24	0.59	−	0.67	3.02
6.90	sabinene	136	C_10_H_16_	984	973	0.14	0.14	6.74	−	5.54	0.45
7.00	*β*-pinene	136	C_10_H_16_	988	980	−	5.34	0.38	−	0.37	1.99
7.69	*α*-terpinene	136	C_10_H_16_	1,025.5	1,018	−	−	4.67	−	10.21	−
7.82	*o*-cymene	134	C_10_H_14_	1,033.9	1,027	0.23	7.93	3.54	−	5.10	0.71
7.90	D-limonene	136	C_10_H_16_	1,039	1,044	6.99	1.98	3.16	**67.67**	3.53	1.52
7.96	eucalyptol	154	C_10_H_18_O	1,042.8	1,046	0.86	0.23	−	−	−	**46.06**
8.37	*γ*-terpinene	136	C_10_H_16_	1,068	1,064	0.33	9.55	8.71	−	17.29	−
8.51	linalool	154	C_10_H_18_O	1,076.3	1,080	−	−	5.77	−	1.70	0.49
8.83	*α*-terpinolene	136	C_10_H_16_	1,094.9	1,097	−	−	2.03	−	3.65	0.39
8.86	limonene oxide	152	C_10_H_16_O	1,096.6	1,119	3.21	−	−	−	−	−
8.98	*p*-menth-2-en-1-ol	154	C_10_H_18_O	1,105	1,117	−	−	**24.86**	−	5.24	−
9.66	camphor	152	C_10_H_16_O	1,159.4	1,150	−	−	−	−	−	26.43
10.05	*p*-menth-1-en-4-ol	154	C_10_H_18_O	1,189	1,180	0.48	0.32	18.68	−	**31.73**	0.68
10.19	*α*-terpineol	154	C_10_H_18_O	1,199.3	1,190	−	−	3.47	−	3.73	1.63
10.24	trans-carveol	152	C_10_H_16_O	1,203.8	1,201	−	3.93	−	−	−	−
10.26	**estragole**	148	C_10_H_12_O	1,205.7	1,196	**84.5**	−	0.30	−	0.44	−
10.78	**cuminaldehyde**	148	C_10_H_12_O	1,254	1,242	−	**49.93**	−	−	−	−
10.82	carvone	150	C_10_H_14_O	1,257.6	1,249	−	−	−	29.72	−	0.93
11.26	anethole	148	C_10_H_12_O	1,296.5	1,301	1.86	−	−	−	−	−
11.29	2-caren-10-al	150	C_10_H_14_O	1,299.1	1,297	−	19.08	0.20	−	−	−
12.72	caryophyllene	204	C_15_H_24_	1,449.8	1,451	−	−	2.93	−	0.92	−
13.39	bicyclogermacrene	204	C_15_H_24_	1,525.9	1,505	−	−	2.09	−	0.77	−

Note. Density of the volatile oils: FEN 934 mg/ml; CUMIN 929 mg/ml; MAJO 866 mg/ml; CAR 871 mg/ml; ANIS 887 mg/ml; LAV 903 mg/ml; −, not detected. FEN, fennel volatile oil; CUMIN, cumin volatile oil; MAJO, marjoram volatile oil; CAR, caraway volatile oil; ANIS, anise volatile oil; LAV, lavender volatile oil. Kovat’s index exp., experimental Kovat’s retention index calculated based on hydrocarbon mixture; Kovat’s index ref., reference Kovat’s retention index based on literature. Bold values represent the major components detected in respective volatile oil sample. Estragole in active FEN and cuminaldehyde in active CUMIN volatile oils are highlighted.

Cumin volatile oil (CUMIN) revealed high content of cuminaldehyde (49.9%), 2-caren-10-al (19.1%), and *γ*-terpinene (9.6%) (Supplementary Figure S2). The content of the volatile oil was in agreement with the literature, while a higher amount of cuminaldehyde and carenal was detected compared to cumin from Syria (Rihawy et al., 2014). Interestingly, anti-infectious properties were reported to be attributed to the presence of cumin aldehyde compounds (cuminaldehyde, 2-caren-10-al) as well as beta-pinene (Rihawy et al., 2014).

Marjoram (= majorana) volatile oil (MAJO) revealed the presence of *p*-menth-2-en-1-ol (24.9%), *p*-menth-1-en-4-ol (syn. terpinen-4-ol) (18.7%), and *γ*-terpinene (8.7%) (Supplementary Figure S3). In a previous report, *p*-menth-1-en-4-ol was predominant, followed by *γ*-terpinene (Lagha et al., 2019).

Caraway volatile oil (CAR) was rich in D-limonene (67.7%) and carvone (29.7%), the only detected major components (Supplementary Figure S4), which correlated well with the literature (Iacobellis et al., 2005).

The major volatile of anise oil (ANIS) was *p*-menth-1-en-4-ol (31.7%), while *γ*-terpinene (17.3%) and *α*-terpinene (10.2%) were also abundant (Supplementary Figure S5). However, most of the reports refer to trans-anethole as a major component (Tepe et al., 2006).

Lavender volatile oil (LAV) revealed eucalyptol (syn. 1,8-cineole) (46.1%) as the major component (Supplementary Figure S6) similar to a previous report (Murbach Teles Andrade et al., 2014), while the content of camphor (26.4%) was higher in comparison with the other lavender samples (Bialon et al., 2019)

Although plant species possess a genetic predisposition to produce certain secondary metabolites, the variations in the total content of these metabolites occur at different levels and under various conditions. Thus, the expression of the chemical content of the volatile oils may change due to various biochemical, physiological, ecological, and evolutionary interactions (Murbach Teles Andrade et al., 2014).

## Discussion

Fennel, cumin, marjoram, lavender, caraway, and anise are the common herbs widely used by the Mediterranean nations in their diet, therapeutics, and cosmetics. Their hydrodistillated volatile oils were screened for various biological activities, including anti-inflammatory, anti-allergic, antimicrobial, and antiviral activities. Several oils showed antimicrobial and anti-inflammatory potential. Their effect on the signaling pathways in human neutrophils was also explored.

In the last three decades, microbial infections have emerged as a major threat to the global health care system with skyrocketing incidences of microbial resistance to available antibiotics. Large pharmaceutical companies stopped investing in antibiotic research because of the rapid emergence of microbial resistance due to antibiotic misuse. Health care authorities encourage scientists all over the world to take the lead and come up with new antimicrobial agents especially from natural sources with potent activity and high safety index. Volatile oils are among the most interesting agents that can be safely used to tackle microbial infections because of their long history of safe use. Aiming to reveal the scientific basis of volatile oil uses as antimicrobial agents, we evaluated the antimicrobial activity of some of the most used volatile oils. Marjoram was the most effective against gram-negative *E. coli* and *B. subtilis*, and gram-positive *V. harveyi* with a minimal inhibitory concentration of 128–256 μg/ml. The most sensitive strain to cumin, anise, and lavender volatile oils was the gram-positive *B. subtilis*. In general, the gram-positive bacteria were found to be more sensitive to natural products compared with the gram-negative species (Silva and Fernandes Júnior, 2010). Our results demonstrate a mixed sensitivity of the tested volatile oils to both gram-positive and gram-negative bacteria using submilligram per milliliter concentrations. Previous reports have demonstrated the antimicrobial effect of fennel, marjoram, and lavender volatile oils against *S. aureus* and *E. coli* at higher concentrations (MIC_50_ 2.37–21.3 mg/ml) in strains isolated from human clinical cases (Murbach Teles Andrade et al., 2014) or at lower concentrations (MIC_50_ 0.25–0.5 mg/ml) in cultured strains (Hammer et al., 1999), while all volatile oils were inactive against *P. aeruginosa*. We observed similar results for marjoram (MIC_50_ 0.125–0.25 mg/ml) in the cultured strains. We additionally tested the antimicrobial activity of cumin and caraway volatile oils. Cumin demonstrated moderate antimicrobial effect against gram-negative *E. coli* and *V. harveyi*, and gram-positive *B. subtilis* (MIC 512, 512, and 256 μg/ml, respectively). Cuminaldehyde might be the responsible component for the antimicrobial activity, as its antimicrobial potency has been reported earlier for *E. coli* and *S. aureus*, as well as other microorganisms (De et al., 2003; Monteiro-Neto et al., 2020). Gram-positive *B. subtilis* was the most sensitive bacterial strain to the tested volatile oils, including marjoram, cumin, anise, and lavender, and the results were in good agreement with previous literature (Prabuseenivasan et al., 2006). *E. coli* (Anand and Griffiths, 2003) and *V. harveyi* (Zhang et al., 2020) cause complicated infections in humans and fish, while *B. subtilis* serves as a probiotic (Olmos et al., 2020). Thus, the antimicrobial effect against *B. subtilis* revealed a limitation to the use of these volatile oils in the diet. Our data pointed out that *C. albicans* was not affected by the volatile oils treatment, and according to previous literature, a higher concentration of volatile oils might be required to affect this fungus (Singh et al., 2002; Bozin et al., 2006; He et al., 2019).

The immune system represents the most complicated networking structure in the human body, and its malfunction requires potential immunotherapeutic agents for treating specific immune and the associated chronic diseases (Grivennikov et al., 2010). Also, in response to infection, a proper regulation of inflammatory processes is needed to maintain the body's homeostasis and health. Unfortunately, there is a deficiency in the safe and effective treatments for neutrophilic inflammatory diseases, such as COPD (Wright et al., 2016), psoriasis (Chiang et al., 2019), life-threatening sepsis (Bone, 1992), or coronavirus-associated ARDS (Chiang et al., 2020). This suggests the urgent need to search for new neutrophil-suppressing agents to improve the treatment of neutrophilic-related inflammatory diseases, including infection-associated ARDS and lung injury. Human neutrophils serve as the first-line in fending off pathogens. The neutrophil defensive features include respiratory burst with the generation of ROS and superoxide, degranulation with the release of antimicrobial peptides and elastase, as well as NETs formation. However, exaggerated reactions lead to many acute, chronic, and also autoimmune neutrophilic inflammatory disorders, such as ARDS, a severe complication of COVID-19 leading to cytokine storm and even death of the infected patients (Abdin et al., 2020). Up to date, COVID-associated ARDS lacks potential treatment; however, drugs targeting neutrophils were suggested as a potential treatment (Chiang et al., 2020).

The fennel raw material is very cheap (around ∼2 $/kg) (Lubbe and Verpoorte, 2011), and thus holds an opportunity for further development. Reports on the anti-inflammatory activity of fennel and cumin and their major components are scarce. The fennel fruit extract, but not its volatile oil, was previously reported to inhibit acute and subacute inflammatory diseases and type IV allergic reactions (Choi and Hwang, 2004). Another study demonstrated the anti-allergic and anti-inflammatory activities of certain volatile oils including lemongrass, sandalwood, chamomile using *in vitro* and *in vivo* models (Mitoshi et al., 2014). In that study, Mitoshi et al. reported marjoram as inactive, while caraway as showing a weak inhibitory effect on the degranulation of RBL-2H3 mast cells. Cumin also inhibited both degranulation in RBL-2H3 cells and TNF-α production in RAW264.7 murine macrophages. We found that none of the volatile oils inhibited the degranulation in RBL-2H3 at 100 μg/ml, which may be attributed to different experimental procedures. Hence, higher concentrations of volatile oils may be needed to observe the anti-allergic effects.

Fennel (*F. vulgare*) and cumin (*C. cyminum*) fruits' volatile oils significantly suppressed the activation of human neutrophils, including respiratory burst and degranulation induced by the FPR agonists fMLF/CB (FPR1) and MMK1 (FPR2) in human neutrophils. The fennel volatile oil was particularly strong in preventing the fMLF-induced superoxide generation (IC_50_ 3.46 μg/ml). The cytotoxicity and free-radical scavenging effects (ABTS and DPPH) or the direct inhibition of elastase did not account for the observed effects, which indicates to the ability of fennel and cumin volatile oils to suppress the function of neutrophils. Interestingly, a major component of black cumin (*Nigella sativa*) seeds' volatile oil, thymoquinone, was reported to inhibit fMLF-induced superoxide production, the release of myeloperoxidase, and exocytosis of specific and azurophilic granules as evidenced by the suppression of cell surface expression of gp91PHOX and CD11b in neutrophils (Boudiaf et al., 2016). In agreement with our results, the inhibition of superoxide anion generation by thymoquinone was not due to radical scavenging effects (using xanthine/xanthine oxidase assay). Both fennel and cumin significantly shortened calcium influx recovery time and suppressed the phosphorylation of MAPK, including ERK, JNK, and p38 phosphorylation, with downstream signaling leading to the activation of respiratory burst and degranulation in human neutrophils. According to previous studies, cumin oil exerted anti-inflammatory effects in LPS-stimulated RAW macrophages through inhibiting NF-κB and MAPK (Wei et al., 2015). That bodes well with our results, indicating the suppressive effect of cumin and fennel volatile oils on the downstream signaling pathways of neutrophils and other leukocytes. Interestingly, a protein kinase (PKA) inhibitor H89 partially restored the superoxide anion generation inhibited by both FEN and CUMIN, which together with the inhibitory effect on calcium influx recovery time indicated a role of the cAMP/PKA pathway in the anti-inflammatory effects of FEN and CUMIN.

Furthermore, it would be worth understanding whether a single component or a natural well-prepared mixture is responsible for the observed effects in CUMIN and FEN. According to the literature, anethole is the most common major component detected in fennel volatile oils and has been reported to show anti-inflammatory effects *in vivo* using the chronic lung inflammation (COPD) model (reduction of TNF-α and IL-6) (Kim et al., 2017) and the lipopolysaccharide-induced acute lung injury model (decreased neutrophils and macrophages numbers, and the inflammatory mediators MMP-9, TNF-α, and NO, as well as the NF-кB pathway) (Kang et al., 2013). According to our results, the major identified component of fennel oil was estragole. Estragole was reported to exhibit anti-inflammatory effects *in vitro* (over 200 μM) in LPS-induced RAW 264.7 cells on NO production (Marrelli et al., 2020) by suppressing NF-кB and Nrf-2 pathways (Roy et al., 2018) as well as *in vivo* (30 mg/kg) reduction of paw edema, and leukocyte emigration in the peritoneal fluid (Rodrigues et al., 2016). Cumin contains cuminaldehyde as a major bioactive component (Singh et al., 2021), which was previously found to possess antinociceptive and antineuropathic effects *in vivo* (with involvement of opioid receptors and the L-arginine/NO/cGMP pathway) and anti-inflammatory effects on cyclooxygenase and cytokines. However, its anti-inflammatory function deserves further investigation (Koohsari et al., 2020).

Our results implied the important role of cumin and fennel volatile oils on human neutrophils, the immune cells being involved in a plethora of physiological processes, and human disorders. Thus, the Mediterranean diet rich in cumin and fennel might be a perfect measure to counteract neutrophilic inflammatory diseases. The implementation of well-designed clinical trials is required to determine the effectiveness and safety of these volatile oils as drug leads.

## Conclusion

Our findings shed light on the antimicrobial effect of commonly used volatile oils. Cumin and fennel volatile oils showed a direct effect on human neutrophils, resulting in a suppressive effect on respiratory burst and degranulation with a profound role on FPR receptors (Graphical Abstract). The inhibition of elastase release was comparable to the positive control, genistein. The significant inhibition of calcium and the MAPK and Akt signaling pathways indicate that fennel and cumin volatile oils are capable of effectively altering the function of human neutrophils. Cumin and fennel volatile oils suppressed the activation of neutrophils and might show therapeutic potential for the treatment of neutrophilic inflammatory diseases.

## Data Availability

The raw data supporting the conclusions of this article will be made available by the authors, without undue reservation.

## Supplementary Material

Supplementary Figures S1 to S6 include details on GC-MS analysis of each volatile oil sample.

## Abbreviations

ABTS, 2,2'-azino-bis(3-ethylbenzothiazoline-6-sulphonic acid; ANIS, anise volatile oil; CAR, caraway volatile oil; CB, cytochalasin B; CUMIN, cumin volatile oil; DPPH, 2,2-diphenyl-1-picrylhydrazyl; DMEM, Dulbecco’s modified Eagle’s medium; FBS, fetal bovine serum; FEN, fennel volatile oil; FPR, formyl peptide receptor; LAV, lavender volatile oil; MAJO, marjoram volatile oil; MAPK, mitogen-activated protein kinases; MTT, [3-(4,5-dimethylthiazol-2-yl)-2,5-diphenyltetrazolium bromide]; SOD, superoxide dismutase.
