# Supplementary material for: Anti-Inflammatory and Antimicrobial Volatile Oils: Fennel and Cumin Inhibit Neutrophilic Inflammation via Regulating Calcium and MAPKs
Source: Front Pharmacol. 2021 Oct 11;12:674095. doi: 10.3389/fphar.2021.674095 (PMC8545060; doi:10.3389/fphar.2021.674095)
Supplement: Supplementary file 1 [file DataSheet1.doc]

**Supplementary material**

# Anti-inflammatory and anti-microbial volatile oils: Fennel and cumin inhibit neutrophilic inflammation via regulating calcium and MAPKs.

**Michal Korinek^1,2,3^, Heba Handoussa^4*^, Yi-Hong Tsai^1^, You-Ying Chen^5^, Meng-Hua Chen^2^, Zan-Wei Chiou^2^, Yu Fang^2^, Fang-Rong Chang^1^, Chia-Hung Yen^1^, Chung-Fan Hsieh^6^, Bing-Hung Chen^3^, Mohamed El-Shazly^4,7*^, Tsong-Long Hwang^2,8,9,10*^**

General conditions of GC-MS analysis and Supplementary Figures S1 to S6 (p. S2-S17) that include details on GC-MS analysis of each volatile oil sample.

General conditions of GC-MS. p. S1

**Figure S1.** GC-MS analysis of fennel volatile oil (FEN), density 934 mg/mL. p. S2-S3

**Figure S2.** GC-MS analysis of cumin volatile oil (CUMIN), density 929 mg/mL. p. S4-S6

**Figure S3.** GC-MS analysis of marjoram volatile oil (MAJO), density 866 mg/mL. p. S7-S12

**Figure S4.** GC-MS analysis of caraway volatile oil (CAR), density 871 mg/mL. p. S13-S14

**Figure S5.** GC-MS analysis of anise volatile oil (ANIS), density 887 mg/mL. p. S15-S18

**Figure S6.** GC-MS analysis of lavender volatile oil (LAV), density 903 mg/mL p. S19-S20

**GC-MS analysis data, general condition.**

**ThermoFinnigan (** *TRACE***GC -** *POLARIS***Q)**

**GC** Inlet Temp. 250 °C He Gas Carrier Flow 1.0 ml/min.

Column: **GsBP-5MS** **30m*0.25mm*0.50μm**

Oven Ramp:

(1) Initial--- 75 °C (0-5min.)

(2) Ramp--- Rate 15 °C/min. (5-20min.; 75-300°C), and hold 300 °C. (after 20min.)

**EI-MS 70 eV**

(Ion Source Temp. 230 °C)

**Figure S1. GC-MS analysis of fennel volatile oil (FEN), density 934 mg/mL.**

##

| **Retention Time (min)** | **Name** | **% Area Relative** | **Mw** | **Formula** | **Kovat’s index exp.^a^** | **Kovat’s index ref.^b^** | **Fragmentation** | **Reference^c^** | **NIST GC-MS LINK^c^** | **Reference Column^c^** | **CAS number** |
| --- | --- | --- | --- | --- | --- | --- | --- | --- | --- | --- | --- |
| **fennel volatile oil (FEN), density 934 mg/mL** | | | | | | | | | | | |
| 6.09 | 𝛼-pinene | 0.67 | 136 | C_10_H_16_ | 948 | 943 | 136, 121, 105, 93, 91, 77 | El-Massry et al., Food Chem., 2002, 79, 3, 331-336, <https://doi.org/10.1016/S0308-8146(02)00164-4> | [LINK](https://webbook.nist.gov/cgi/cbook.cgi?ID=C80568&Units=SI&Mask=2200) | DB-5 | 80-56-8 |
| 6.9 | sabinene | 0.14 | 136 | C_10_H_16_ | 984 | 973 | 136, 121, 107, 93, 91, 79, 77 | Hazzit et al., J. Agric. Food Chem., 2006, 54, 17, 6314-6321, <https://doi.org/10.1021/jf0606104> | [LINK](https://webbook.nist.gov/cgi/cbook.cgi?Name=Sabinene&Units=SI&cMS=on&cGC=on) | HP-5MS | 3387-41-5 |
| 7.82 | *o*-cymene | 0.23 | 134 | C_10_H_14_ | 1033.9 | 1027 | 134, 119, 117, 91 | Lucero et al., J. Essent. Oil Res., 2006, 18, 2, 121-125, <https://doi.org/10.1080/10412905.2006.9699039> | [LINK](https://webbook.nist.gov/cgi/cbook.cgi?Name=o-cymene&Units=SI&cMS=on&cGC=on#ref-17) | DB-5 | 527-84-4 |
| 7.9 | D-limonene | 6.99 | 136 | C_10_H_16_ | 1039 | 1044 | 136, 121, 107, 93, 91, 79, 77, 67 | Hazzit et al., J. Agric. Food Chem., 2006, 54, 17, 6314-6321, <https://doi.org/10.1021/jf0606104> | [LINK](https://webbook.nist.gov/cgi/cbook.cgi?ID=C138863&Units=SI&Mask=2200#ref-21) | HP-5MS | 138-86-3 |
| 7.96 | eucalyptol  (syn. 1,8-cineol) | 0.86 | 154 | C_10_H_18_O | 1042.8 | 1046 | 154, 139, 125, 111, 93, 81 | Hazzit et al., J. Agric. Food Chem., 2006, 54, 17, 6314-6321, <https://doi.org/10.1021/jf0606104> | [LINK](https://webbook.nist.gov/cgi/cbook.cgi?Name=eucalyptol&Units=SI&cMS=on&cGC=on) | HP-5MS | 470-82-6 |
| 8.37 | γ-terpinene | 0.33 | 136 | C_10_H_16_ | 1068 | 1064 | 136, 121, 107, 105, 93, 91, 77 | Kartal et al., Food Chem., 2007, 100, 2, 584-589, https://doi.org/10.1016/j.foodchem.2005.09.084 | [LINK](https://webbook.nist.gov/cgi/cbook.cgi?Name=γ-terpinene&Units=SI&cMS=on&cGC=on#ref-10) | HP-5MS | 99-85-4 |
| 8.86 | limonene oxide | 3.21 | 152 | C_10_H_16_O | 1096.6 | 1119 | 152, 137, 109, 81, 79, 67, 53 | MacLeod et al., Phytochemistry, 1988, 27, 7, 2185-2188, <https://doi.org/10.1016/0031-9422(88)80123-7> | [LINK](https://webbook.nist.gov/cgi/cbook.cgi?Name=limonene+oxide&Units=SI&cMS=on&cGC=on#ref-2) | BP-1 | 1195-92-2 |
| 10.05 | terpinen-4-ol  (syn. *p*-ment-1-en-4-ol) | 0.48 | 154 | C_10_H_18_O | 1189 | 1180 | 154, 136, 121, 111, 93, 91, 77 | Maia et al., Flavour Fragr. J., 2005, 20, 5, 474-477, <https://doi.org/10.1002/ffj.1499> | [LINK](https://webbook.nist.gov/cgi/cbook.cgi?Name=terpinen-4-ol&Units=SI&cMS=on&cGC=on) | DB-5MS | 877467-48-6 |
| 10.26 | **estragole** | **84.5** | 148 | C_10_H_12_O | 1205.7 | 1196 | 148, 147, 133, 121, 117, 115, 105, 91, 77 | Tepe et al., Food Chem., 2006, 97, 4, 719-724, <https://doi.org/10.1016/j.foodchem.2005.05.045> | [LINK](https://webbook.nist.gov/cgi/cbook.cgi?Name=estragole&Units=SI&cMS=on&cGC=on) | HP-5MS | 140-67-0 |
| 11.26 | **anethole** | **1.86** | 148 | C_10_H_12_O | 1296.5 | 1301 | 148, 147, 133, 121, 117, 115, 105, 91, 77 | Tepe et al., Food Chem., 2006, 97, 4, 719-724, <https://doi.org/10.1016/j.foodchem.2005.05.045> | [LINK](https://webbook.nist.gov/cgi/cbook.cgi?ID=C4180238&Units=SI&Mask=2200#ref-2) | HP-5MS | 4180-23-8 |

^a^Kovat’s index (KI) exp., experimental Kovat’s retention index calculated based on hydrocarbon mixture set as isothermic with logs (GsBP-5MS; 30m*0.25mm*0.50um; 15 °C/min to 300°C)

^b^Kovat’s index ref., reference Kovat’s retention index based on literature according to NIST Chemistry WebBook (SRD 69; July 2021).

^c^Reference literature and link to National Institute of Standards and Technology (NIST) is provided, https://webbook.nist.gov. The reference column condition: Kovats' RI non-polar column, temperature ramp (upon availability).

Bold values represent the major or important components detected in the volatile oil sample. Mw, molecular weight.

**Figure S2. GC-MS analysis of cumin volatile oil (CUMIN), density 929 mg/mL.**

| **Retention Time (min)** | **Name** | **% Area Relative** | **Mw** | **Formula** | **Kovat’s index exp.^a^** | **Kovat’s index ref.^b^** | **Fragmentation** | **Reference^c^** | **NIST GC-MS LINK^c^** | **Reference Column^c^** | **CAS number** |
| --- | --- | --- | --- | --- | --- | --- | --- | --- | --- | --- | --- |
| **cumin volatile oil (CUMIN), density 929 mg/mL** | | | | | | | | | | | |
| **6.09CUMIN, density 929 mg/mL** | 𝛼-pinene | 0.24 | 136 | C_10_H_16_ | 948 | 943 | 136, 121, 105, 93, 91, 77 | El-Massry et al., Food Chem., 2002, 79, 3, 331-336, <https://doi.org/10.1016/S0308-8146(02)00164-4> | [LINK](https://webbook.nist.gov/cgi/cbook.cgi?ID=C80568&Units=SI&Mask=2200) | DB-5 | 80-56-8 |
| 6.90 | sabinene | 0.14 | 136 | C_10_H_16_ | 984 | 973 | 136, 121, 107, 93, 91, 79, 77 | Hazzit et al., J. Agric. Food Chem., 2006, 54, 17, 6314-6321, <https://doi.org/10.1021/jf0606104> | [LINK](https://webbook.nist.gov/cgi/cbook.cgi?Name=Sabinene&Units=SI&cMS=on&cGC=on) | HP-5MS | 3387-41-5 |
| 7.00 | β-pinene | 5.34 | 136 | C_10_H_16_ | 988 | 980 | 136, 121, 91, 77 | Adams et al., Biochem. Syst. Ecol., 2006, 34, 3, 205-211, https://doi.org/10.1016/j.bse.2005.11.004 | [LINK](https://webbook.nist.gov/cgi/cbook.cgi?Name=beta-pinene&Units=SI&cMS=on&cGC=on#ref-13) | DB-5 | 127-91-3 |
| 7.82 | *o*-cymene | 7.93 | 134 | C_10_H_14_ | 1033.9 | 1027 | 134, 119, 117, 91 | Lucero et al., J. Essent. Oil Res., 2006, 18, 2, 121-125, <https://doi.org/10.1080/10412905.2006.9699039> | [LINK](https://webbook.nist.gov/cgi/cbook.cgi?Name=o-cymene&Units=SI&cMS=on&cGC=on#ref-17) | DB-5 | 527-84-4 |
| 7.90 | D-limonene | 1.98 | 136 | C_10_H_16_ | 1039 | 1044 | 136, 121, 107, 93, 91, 79, 77, 67 | Hazzit et al., J. Agric. Food Chem., 2006, 54, 17, 6314-6321, <https://doi.org/10.1021/jf0606104> | [LINK](https://webbook.nist.gov/cgi/cbook.cgi?ID=C138863&Units=SI&Mask=2200#ref-21) | HP-5MS | 138-86-3 |
| 7.95 | eucalyptol (syn. 1,8-cineol) | 0.23 | 154 | C_10_H_18_O | 1042.8 | 1046 | 154, 139, 125, 111, 93, 81 | Hazzit et al., J. Agric. Food Chem., 2006, 54, 17, 6314-6321, <https://doi.org/10.1021/jf0606104> | [LINK](https://webbook.nist.gov/cgi/cbook.cgi?Name=eucalyptol&Units=SI&cMS=on&cGC=on) | HP-5MS | 470-82-6 |
| 8.37 | γ-terpinene | 9.55 | 136 | C_10_H_16_ | 1068 | 1064 | 136, 121, 107, 105, 93, 91, 77 | Kartal et al., Food Chem., 2007, 100, 2, 584-589, <https://doi.org/10.1016/j.foodchem.2005.09.084> | [LINK](https://webbook.nist.gov/cgi/cbook.cgi?Name=γ-terpinene&Units=SI&cMS=on&cGC=on#ref-10) | HP-5MS | 99-85-4 |
| 10.05 | terpinen-4-ol (syn. *p*-ment-1-en-4-ol) | 0.32 | 154 | C_10_H_18_O | 1189 | 1180 | 154, 136, 121, 111, 93, 91, 77 | Maia et al., Flavour Fragr. J., 2005, 20, 5, 474-477, <https://doi.org/10.1002/ffj.1499> | [LINK](https://webbook.nist.gov/cgi/cbook.cgi?Name=terpinen-4-ol&Units=SI&cMS=on&cGC=on) | DB-5MS | 877467-48-6 |
| 10.24 | trans-carveol | 3.93 | 152 | C_10_H_16_O | 1203.8 | 1201 | 152, 121, 109, 91, 81, 79, 77, 67 | Gkinis et al., Z. Naturforsch. C, 2003, 58, 681-686, <https://doi.org/10.1515/znc-2003-9-1015> | [LINK](https://webbook.nist.gov/cgi/cbook.cgi?Name=trans-carveol&Units=SI&cMS=on&cGC=on#ref-10) | HP-5MS | 1197-07-5 |
| 10.78 | **cuminaldehyde** | **49.93** | 148 | C_10_H_12_O | 1254 | 1242 | 148, 133, 119, 105, 91, 77 | Adams et al., Biochem. Syst. Ecol., 2005, 33, 8, 771-787, <https://doi.org/10.1016/j.bse.2005.01.001> | [LINK](https://webbook.nist.gov/cgi/cbook.cgi?Name=cuminaldehyde&Units=SI&cMS=on&cGC=on) | DB-5 | 122-03-2 |
| 11.29 | 2-caren-10-al | 19.08 | 150 | C_10_H_14_O | 1299.1 | 1297 | 150, 135, 121, 107, 91, 79, 77 | Ghasemi et al., Nat Prod Res., 2020, 34, 6, 843-846, <https://doi.org/10.1080/14786419.2018.1501686> | [LINK](https://pubchem.ncbi.nlm.nih.gov/compound/2-Caren-10-al#section=WIPO-PATENTSCOPE) | HP-5MS | NA |

^a^Kovat’s index (KI) exp., experimental Kovat’s retention index calculated based on hydrocarbon mixture set as isothermic with logs (GsBP-5MS; 30m*0.25mm*0.50um; 15 °C/min to 300°C)

^b^Kovat’s index ref., reference Kovat’s retention index based on literature according to NIST Chemistry WebBook (SRD 69; July 2021).

^c^Reference literature and link to National Institute of Standards and Technology (NIST) is provided, https://webbook.nist.gov. The reference column condition: Kovats' RI non-polar column, temperature ramp (upon availability).

Bold value represents the major component detected in the volatile oil sample. NA, not available. Mw, molecular weight.

**Figure S3. GC-MS analysis of marjoram volatile oil (MAJO), density 866 mg/mL.**

| **Retention Time (min)** | **Name** | **% Area Relative** | **Mw** | **Formula** | **Kovat’s index exp.^a^** | **Kovat’s index ref.^b^** | **Fragmentation** | **Reference^c^** | **NIST GC-MS LINK^c^** | **Reference Column^c^** | **CAS number** |
| --- | --- | --- | --- | --- | --- | --- | --- | --- | --- | --- | --- |
| **marjoram volatile oil (MAJO), density 866 mg/mL** | | | | | | | | | | | |
| **6.09CUMIN, density 929 mg/mL** | 𝛼-pinene | 0.59 | 136 | C_10_H_16_ | 948 | 943 | 136, 121, 105, 93, 91, 77 | El-Massry et al., Food Chem., 2002, 79, 3, 331-336, <https://doi.org/10.1016/S0308-8146(02)00164-4> | [LINK](https://webbook.nist.gov/cgi/cbook.cgi?ID=C80568&Units=SI&Mask=2200) | DB-5 | 80-56-8 |
| 6.90 | sabinene | 6.74 | 136 | C_10_H_16_ | 984 | 973 | 136, 121, 93, 91, 79, 77 | Hazzit et al., J. Agric. Food Chem., 2006, 54, 17, 6314-6321, <https://doi.org/10.1021/jf0606104> | [LINK](https://webbook.nist.gov/cgi/cbook.cgi?Name=Sabinene&Units=SI&cMS=on&cGC=on) | HP-5MS | 3387-41-5 |
| 7.00 | β-pinene | 0.38 | 136 | C_10_H_16_ | 988 | 980 | 136, 121, 91, 77 | Adams et al., Biochem. Syst. Ecol., 2006, 34, 3, 205-211, <https://doi.org/10.1016/j.bse.2005.11.004> | [LINK](https://webbook.nist.gov/cgi/cbook.cgi?Name=beta-pinene&Units=SI&cMS=on&cGC=on#ref-13) | DB-5 | 127-91-3 |
| 7.69 | α-terpinene | 4.67 | 136 | C_10_H_16_ | 1025.5 | 1018 | 136, 121, 105, 93, 91, 79, 77 | Adams et al., Biochem. Syst. Ecol., 2006, 34, 3, 205-211, <https://doi.org/10.1016/j.bse.2005.11.004> | [LINK](https://webbook.nist.gov/cgi/cbook.cgi?Name=alpha+terpinene&Units=SI&cMS=on&cGC=on#ref-8) | DB-5 | 99-86-5 |
| 7.82 | *o*-cymene | 3.54 | 134 | C_10_H_14_ | 1033.9 | 1027 | 134, 119, 117, 115, 91 | Lucero et al., J. Essent. Oil Res., 2006, 18, 2, 121-125, <https://doi.org/10.1080/10412905.2006.9699039> | [LINK](https://webbook.nist.gov/cgi/cbook.cgi?Name=o-cymene&Units=SI&cMS=on&cGC=on#ref-17) | DB-5 | 527-84-4 |
| 7.90 | D-limonene | 3.16 | 136 | C_10_H_16_ | 1039 | 1044 | 136, 121, 107, 93, 91, 79, 77, 67 | Hazzit et al., J. Agric. Food Chem., 2006, 54, 17, 6314-6321, <https://doi.org/10.1021/jf0606104> | [LINK](https://webbook.nist.gov/cgi/cbook.cgi?ID=C138863&Units=SI&Mask=2200#ref-21) | HP-5MS | 138-86-3 |
| 8.37 | γ-terpinene | 8.71 | 136 | C_10_H_16_ | 1068 | 1064 | 136, 121, 105, 93, 91, 77 | Kartal et al., Food Chem., 2007, 100, 2, 584-589, <https://doi.org/10.1016/j.foodchem.2005.09.084> | [LINK](https://webbook.nist.gov/cgi/cbook.cgi?Name=γ-terpinene&Units=SI&cMS=on&cGC=on#ref-10) | HP-5MS | 99-85-4 |
| 8.51 | linalool | 5.77 | 154 | C_10_H_18_O | 1076.3 | 1080 | 154, 139, 121, 111, 93, 91, 77, 67 | Tepe et al., Food Chem., 2006, 97, 4, 719-724, <https://doi.org/10.1016/j.foodchem.2005.05.045> | [LINK](https://webbook.nist.gov/cgi/cbook.cgi?Name=linalool&Units=SI&cMS=on&cGC=on#ref-27) | HP-5MS | 78-70-6 |
| 8.83 | α-terpinolene (syn. *p*-mentha-1,4,(8)-diene) | 2.03 | 136 | C_10_H_16_ | 1094.9 | 1097 | 136, 121, 105, 93, 91, 79, 77, 65, 51 | Kartal et al., Food Chem., 2007, 100, 2, 584-589, <https://doi.org/10.1016/j.foodchem.2005.09.084> | [LINK](https://webbook.nist.gov/cgi/cbook.cgi?Name=586-62-9&Units=SI&cMS=on&cGC=on#ref-7) | HP-5MS | 586-62-9 |
| 8.98 | ***cis*-*p*-menth-2-en-1-ol** | **24.86** | 154 | C_10_H_18_O | 1105 | 1117 | 154, 139, 136, 121, 111, 93, 91, 79, 77 | Frizzo et al., Flavour Fragr. J., 2001, 16, 4, 286-288, <https://doi.org/10.1002/ffj.998> | [LINK](https://webbook.nist.gov/cgi/cbook.cgi?ID=R77397&Units=SI&Mask=2200) | HP-5 | 29803-82-5 |
| 10.05 | **terpinen-4-ol (syn. *p*-ment-1-en-4-ol)** | **18.68** | 154 | C_10_H_18_O | 1189 | 1180 | 154, 136, 121, 111, 93, 91, 77 | Maia et al., Flavour Fragr. J., 2005, 20, 5, 474-477, <https://doi.org/10.1002/ffj.1499> | [LINK](https://webbook.nist.gov/cgi/cbook.cgi?Name=terpinen-4-ol&Units=SI&cMS=on&cGC=on) | DB-5MS | 877467-48-6 |
| 10.19 | α-terpineol | 3.47 | 154 | C_10_H_18_O | 1199.3 | 1190 | 155, 136, 121, 105, 93, 91, 77 67 | Hazzit et al., J. Agric. Food Chem., 2006, 54, 17, 6314-6321, <https://doi.org/10.1021/jf0606104> | [LINK](https://webbook.nist.gov/cgi/cbook.cgi?ID=C98555&Units=SI&Mask=2200#ref-13) | HP-5MS | 98-55-5 |
| 10.26 | estragole | 0.3 | 148 | C_10_H_12_O | 1296.5 | 1301 | 148, 147, 133, 121, 117, 115, 105, 91, 77 | Tepe et al., Food Chem., 2006, 97, 4, 719-724, <https://doi.org/10.1016/j.foodchem.2005.05.045> | [LINK](https://webbook.nist.gov/cgi/cbook.cgi?Name=estragole&Units=SI&cMS=on&cGC=on) | HP-5MS | 4180-23-8 |
| 11.29 | 2-caren-10-al | 0.3 | 150 | C_10_H_14_O | 1299.1 | 1297 | 150, 135, 121, 107, 91, 79, 77 | Ghasemi et al., Nat Prod Res., 2020, 34, 6, 843-846, <https://doi.org/10.1080/14786419.2018.1501686> | [LINK](https://pubchem.ncbi.nlm.nih.gov/compound/2-Caren-10-al#section=WIPO-PATENTSCOPE) | HP-5MS | NA |
| 12.72 | caryophyllene | 2.93 | 204 | C15H24 | 1449.8 | 1451 | 204, 189, 161, 133, 119, 105, 91, 77, 67 | Tepe et al., Food Chem., 2006, 97, 4, 719-724, <https://doi.org/10.1016/j.foodchem.2005.05.045> | [LINK](https://webbook.nist.gov/cgi/cbook.cgi?Name=caryophyllene&Units=SI&cMS=on&cGC=on) | HP-5MS | 87-44-5 |
| 13.39 | bicyclogermacrene | 2.09 | 204 | C15H24 | 1525.9 | 1505 | 204, 189, 161, 133, 121, 119, 105, 91, 79, 65 | Pripdeevech et al., Chiang Mai J. Sci., 2013, 40, 2, 214-223, <https://www.thaiscience.info/journals/Article/CMJS/10886427.pdf> | [LINK](https://webbook.nist.gov/cgi/cbook.cgi?ID=C67650902&Units=SI&Mask=2200) | HP-5 MS | 67650-90-2 |

^a^Kovat’s index (KI) exp., experimental Kovat’s retention index calculated based on hydrocarbon mixture set as isothermic with logs (GsBP-5MS; 30m*0.25mm*0.50um; 15 °C/min to 300°C)

^b^Kovat’s index ref., reference Kovat’s retention index based on literature according to NIST Chemistry WebBook (SRD 69; July 2021).

^c^Reference literature and link to National Institute of Standards and Technology (NIST) is provided, https://webbook.nist.gov. The reference column condition: Kovats' RI non-polar column, temperature ramp (upon availability).

Bold values represent the major components detected in the volatile oil sample. Mw, molecular weight.

**Figure S4. GC-MS analysis of caraway volatile oil (CAR), density 871 mg/mL** .

| **Retention Time (min)** | **Name** | **% Area Relative** | **Mw** | **Formula** | **Kovat’s index exp.^a^** | **Kovat’s index ref.^b^** | **Fragmentation** | **Reference^c^** | **NIST GC-MS LINK^c^** | **Reference Column^c^** | **CAS number** |
| --- | --- | --- | --- | --- | --- | --- | --- | --- | --- | --- | --- |
| **caraway volatile oil (CAR), density 871 mg/mL** | | | | | | | | | | | |
| **7.90CUMIN, density 929 mg/mL** | **D-limonene** | **67.67** | 136 | C_10_H_16_ | 1039 | 1044 | 136, 121, 107, 93, 91, 79, 77, 67 | Hazzit et al., J. Agric. Food Chem., 2006, 54, 17, 6314-6321, <https://doi.org/10.1021/jf0606104> | [LINK](https://webbook.nist.gov/cgi/cbook.cgi?ID=C138863&Units=SI&Mask=2200#ref-21) | HP-5MS | 138-86-3 |
| 10.82 | carvone | 29.72 | 150 | C_10_H_14_O | 1257.6 | 1249 | 150, 135, 108, 93, 91, 79, 77, 54 | Baranauskiene et al., Flavour Fragr. J., 2005, 20, 5, 492-500, <https://doi.org/10.1002/ffj.1478> | [LINK](https://webbook.nist.gov/cgi/cbook.cgi?ID=C99490&Units=SI&Mask=2200) | BP-5 | 99-49-0 |

^a^Kovat’s index (KI) exp., experimental Kovat’s retention index calculated based on hydrocarbon mixture set as isothermic with logs (GsBP-5MS; 30m*0.25mm*0.50um; 15 °C/min to 300°C)

^b^Kovat’s index ref., reference Kovat’s retention index based on literature according to NIST Chemistry WebBook (SRD 69; July 2021).

^c^Reference literature and link to National Institute of Standards and Technology (NIST) is provided, https://webbook.nist.gov. The reference column condition: Kovats' RI non-polar column, temperature ramp (upon availability).

Bold value represents the major component detected in the volatile oil sample. Mw, molecular weight.

**Figure S5. GC-MS analysis of anise volatile oil (ANIS), density 887 mg/mL.**

| **Retention Time (min)** | **Name** | **% Area Relative** | **Mw** | **Formula** | **Kovat’s index exp.^a^** | **Kovat’s index ref.^b^** | **Fragmentation** | **Reference^c^** | **NIST GC-MS LINK^c^** | **Reference Column^c^** | **CAS number** |
| --- | --- | --- | --- | --- | --- | --- | --- | --- | --- | --- | --- |
| **anise volatile oil (ANIS), density 887 mg/mL** | | | | | | | | | | | |
| **6.09CUMIN, density 929 mg/mL** | 𝛼-pinene | 0.67 | 136 | C_10_H_16_ | 948 | 943 | 136, 121, 105, 93, 91, 77 | El-Massry et al., Food Chem., 2002, 79, 3, 331-336, <https://doi.org/10.1016/S0308-8146(02)00164-4> | [LINK](https://webbook.nist.gov/cgi/cbook.cgi?ID=C80568&Units=SI&Mask=2200) | DB-5 | 80-56-8 |
| 6.90 | sabinene | 5.54 | 136 | C_10_H_16_ | 984 | 973 | 136, 121, 93, 91, 79, 77 | Hazzit et al., J. Agric. Food Chem., 2006, 54, 17, 6314-6321, <https://doi.org/10.1021/jf0606104> | [LINK](https://webbook.nist.gov/cgi/cbook.cgi?Name=Sabinene&Units=SI&cMS=on&cGC=on) | HP-5MS | 3387-41-5 |
| 7.00 | β-pinene | 0.37 | 136 | C_10_H_16_ | 988 | 980 | 136, 121, 91, 77 | Adams et al., Biochem. Syst. Ecol., 2006, 34, 3, 205-211, <https://doi.org/10.1016/j.bse.2005.11.004> | [LINK](https://webbook.nist.gov/cgi/cbook.cgi?Name=beta-pinene&Units=SI&cMS=on&cGC=on#ref-13) | DB-5 | 127-91-3 |
| 7.69 | α-terpinene | 10.21 | 136 | C_10_H_16_ | 1025.5 | 1018 | 136, 121, 105, 93, 91, 79, 77 | Adams et al., Biochem. Syst. Ecol., 2006, 34, 3, 205-211, <https://doi.org/10.1016/j.bse.2005.11.004> | [LINK](https://webbook.nist.gov/cgi/cbook.cgi?Name=alpha+terpinene&Units=SI&cMS=on&cGC=on#ref-8) | DB-5 | 99-86-5 |
| 7.82 | *o*-cymene | 5.10 | 134 | C_10_H_14_ | 1033.9 | 1027 | 134, 119, 117, 115, 91 | Lucero et al., J. Essent. Oil Res., 2006, 18, 2, 121-125, <https://doi.org/10.1080/10412905.2006.9699039> | [LINK](https://webbook.nist.gov/cgi/cbook.cgi?Name=o-cymene&Units=SI&cMS=on&cGC=on#ref-17) | DB-5 | 527-84-4 |
| 7.91 | D-limonene | 3.53 | 136 | C_10_H_16_ | 1039 | 1044 | 136, 121, 107, 91, 79, 77, 67 | Hazzit et al., J. Agric. Food Chem., 2006, 54, 17, 6314-6321, <https://doi.org/10.1021/jf0606104> | [LINK](https://webbook.nist.gov/cgi/cbook.cgi?ID=C138863&Units=SI&Mask=2200#ref-21) | HP-5MS | 138-86-3 |
| 8.37 | γ-terpinene | 17.29 | 136 | C_10_H_16_ | 1068 | 1064 | 136, 121, 105, 93, 91, 77 | Kartal et al., Food Chem., 2007, 100, 2, 584-589, <https://doi.org/10.1016/j.foodchem.2005.09.084> | [LINK](https://webbook.nist.gov/cgi/cbook.cgi?Name=γ-terpinene&Units=SI&cMS=on&cGC=on#ref-10) | HP-5MS | 99-85-4 |
| 8.51 | linalool | 1.70 | 154 | C_10_H_18_O | 1076.3 | 1080 | 154, 139, 121, 111, 93, 91, 77, 67 | Tepe et al., Food Chem., 2006, 97, 4, 719-724, <https://doi.org/10.1016/j.foodchem.2005.05.045> | [LINK](https://webbook.nist.gov/cgi/cbook.cgi?Name=linalool&Units=SI&cMS=on&cGC=on#ref-27) | HP-5MS | 78-70-6 |
| 8.83 | α-terpinolene (syn. *p*-mentha-1,4,(8)-diene) | 3.65 | 136 | C_10_H_16_ | 1094.9 | 1097 | 136, 121, 105, 93, 91, 79, 77, 65, 51 | Kartal et al., Food Chem., 2007, 100, 2, 584-589, <https://doi.org/10.1016/j.foodchem.2005.09.084> | [LINK](https://webbook.nist.gov/cgi/cbook.cgi?Name=586-62-9&Units=SI&cMS=on&cGC=on#ref-7) | HP-5MS | 586-62-9 |
| 8.97 | cis-*p*-menth-2-en-1-ol | 5.24 | 154 | C_10_H_18_O | 1105 | 1117 | 154, 139, 136, 121, 111, 93, 91, 79, 77 | Frizzo et al., Flavour Fragr. J., 2001, 16, 4, 286-288, <https://doi.org/10.1002/ffj.998> | [LINK](https://webbook.nist.gov/cgi/cbook.cgi?ID=R77397&Units=SI&Mask=2200) | HP-5 | 29803-82-5 |
| 10.05 | **terpinen-4-ol (syn. *p*-ment-1-en-4-ol)** | **31.73** | 154 | C_10_H_18_O | 1189 | 1180 | 154, 136, 121, 111, 93, 91, 77, 71, 67 | Maia et al., Flavour Fragr. J., 2005, 20, 5, 474-477, <https://doi.org/10.1002/ffj.1499> | [LINK](https://webbook.nist.gov/cgi/cbook.cgi?Name=terpinen-4-ol&Units=SI&cMS=on&cGC=on) | DB-5MS | 877467-48-6 |
| 10.2 | α-terpineol | 3.73 | 154 | C_10_H_18_O | 1200 | 1190 | 155, 136, 121, 105, 93, 91, 77 67 | Hazzit et al., J. Agric. Food Chem., 2006, 54, 17, 6314-6321, <https://doi.org/10.1021/jf0606104> | [LINK](https://webbook.nist.gov/cgi/cbook.cgi?ID=C98555&Units=SI&Mask=2200#ref-13) | HP-5MS | 98-55-5 |
| 10.26 | estragole | 0.44 | 148 | C_10_H_12_O | 1205.7 | 1196 | 148, 147, 133, 121, 117, 115, 105, 91, 77 | Tepe et al., Food Chem., 2006, 97, 4, 719-724, <https://doi.org/10.1016/j.foodchem.2005.05.045> | [LINK](https://webbook.nist.gov/cgi/cbook.cgi?Name=estragole&Units=SI&cMS=on&cGC=on) | HP-5MS | 140-67-0 |
| 12.72 | caryophyllene | 0.92 | 204 | C15H24 | 1449.8 | 1451 | 204, 189, 161, 133, 119, 105, 91, 77, 67 | Tepe et al., Food Chem., 2006, 97, 4, 719-724, <https://doi.org/10.1016/j.foodchem.2005.05.045> | [LINK](https://webbook.nist.gov/cgi/cbook.cgi?Name=caryophyllene&Units=SI&cMS=on&cGC=on) | HP-5MS | 87-44-5 |
| 13.39 | bicyclogermacrene | 0.77 | 204 | C15H24 | 1525.9 | 1505 | 204, 189, 161, 133, 121, 119, 105, 91, 79, 65 | Pripdeevech et al., Chiang Mai J. Sci., 2013, 40, 2, 214-223, <https://www.thaiscience.info/journals/Article/CMJS/10886427.pdf> | [LINK](https://webbook.nist.gov/cgi/cbook.cgi?ID=C67650902&Units=SI&Mask=2200) | HP-5 MS | 67650-90-2 |

^a^Kovat’s index (KI) exp., experimental Kovat’s retention index calculated based on hydrocarbon mixture set as isothermic with logs (GsBP-5MS; 30m*0.25mm*0.50um; 15 °C/min to 300°C)

^b^Kovat’s index ref., reference Kovat’s retention index based on literature according to NIST Chemistry WebBook (SRD 69; July 2021).

^c^Reference literature and link to National Institute of Standards and Technology (NIST) is provided, https://webbook.nist.gov. The reference column condition: Kovats' RI non-polar column, temperature ramp (upon availability).

Bold value represents the major component detected in the volatile oil sample. Mw, molecular weight.

**Figure S6. GC-MS analysis of lavender volatile oil (LAV), density 903 mg/mL.**

| **Retention Time (min)** | **Name** | **% Area Relative** | **Mw** | **Formula** | **Kovat’s index exp.^a^** | **Kovat’s index ref.^b^** | **Fragmentation** | **Reference^c^** | **NIST GC-MS LINK^c^** | **Reference Column^c^** | **CAS number** |
| --- | --- | --- | --- | --- | --- | --- | --- | --- | --- | --- | --- |
| **lavender volatile oil (LAV), density 903 mg/mL** | | | | | | | | | | | |
| **6.08CUMIN, density 929 mg/mL** | 𝛼-pinene | 3.02 | 136 | C_10_H_16_ | 948 | 943 | 136, 121, 105, 93, 91, 77 | El-Massry et al., Food Chem., 2002, 79, 3, 331-336, <https://doi.org/10.1016/S0308-8146(02)00164-4> | [LINK](https://webbook.nist.gov/cgi/cbook.cgi?ID=C80568&Units=SI&Mask=2200) | DB-5 | 80-56-8 |
| 6.90 | sabinene | 0.45 | 136 | C_10_H_16_ | 984 | 973 | 136, 121, 93, 91, 79, 77 | Hazzit et al., J. Agric. Food Chem., 2006, 54, 17, 6314-6321, <https://doi.org/10.1021/jf0606104> | [LINK](https://webbook.nist.gov/cgi/cbook.cgi?Name=Sabinene&Units=SI&cMS=on&cGC=on) | HP-5MS | 3387-41-5 |
| 7.00 | β-pinene | 1.99 | 136 | C_10_H_16_ | 988 | 980 | 136, 121, 107, 91, 77 | Adams et al., Biochem. Syst. Ecol., 2006, 34, 3, 205-211, <https://doi.org/10.1016/j.bse.2005.11.004> | [LINK](https://webbook.nist.gov/cgi/cbook.cgi?Name=beta-pinene&Units=SI&cMS=on&cGC=on#ref-13) | DB-5 | 127-91-3 |
| 7.82 | *o*-cymene | 0.71 | 134 | C_10_H_14_ | 1033.9 | 1027 | 134, 119, 117, 115, 91 | Lucero et al., J. Essent. Oil Res., 2006, 18, 2, 121-125, <https://doi.org/10.1080/10412905.2006.9699039> | [LINK](https://webbook.nist.gov/cgi/cbook.cgi?Name=o-cymene&Units=SI&cMS=on&cGC=on#ref-17) | DB-5 | 527-84-4 |
| 7.90 | D-limonene | 1.52 | 136 | C_10_H_16_ | 1039 | 1044 | 136, 121, 107, 91, 79, 77, 67 | Hazzit et al., J. Agric. Food Chem., 2006, 54, 17, 6314-6321, <https://doi.org/10.1021/jf0606104> | [LINK](https://webbook.nist.gov/cgi/cbook.cgi?ID=C138863&Units=SI&Mask=2200#ref-21) | HP-5MS | 138-86-3 |
| 7.96 | **eucalyptol (syn. 1,8-cineol)** | **46.06** | 154 | C_10_H_18_O | 1042.8 | 1046 | 154, 139, 125, 111, 93, 81 | Hazzit et al., J. Agric. Food Chem., 2006, 54, 17, 6314-6321, <https://doi.org/10.1021/jf0606104> | [LINK](https://webbook.nist.gov/cgi/cbook.cgi?Name=eucalyptol&Units=SI&cMS=on&cGC=on) | HP-5MS | 470-82-6 |
| 8.51 | linalool | 0.49 | 154 | C_10_H_18_O | 1076.3 | 1080 | 154, 139, 121, 111, 93, 91, 77, 67 | Tepe et al., Food Chem., 2006, 97, 4, 719-724, <https://doi.org/10.1016/j.foodchem.2005.05.045> | [LINK](https://webbook.nist.gov/cgi/cbook.cgi?Name=linalool&Units=SI&cMS=on&cGC=on#ref-27) | HP-5MS | 78-70-6 |
| 8.83 | α-terpinolene (syn. *p*-mentha-1,4,(8)-diene) | 0.39 | 136 | C_10_H_16_ | 1094.9 | 1097 | 136, 121, 105, 93, 91, 79, 77, 65, 51 | Kartal et al., Food Chem., 2007, 100, 2, 584-589, <https://doi.org/10.1016/j.foodchem.2005.09.084> | [LINK](https://webbook.nist.gov/cgi/cbook.cgi?Name=586-62-9&Units=SI&cMS=on&cGC=on#ref-7) | HP-5MS | 586-62-9 |
| 9.66 | camphor | 26.43 | 152 | C_10_H_16_O | 1159.4 | 1150 | 152, 137, 108, 95, 81, 67 | El-Massry et al., Food Chem., 2002, 79, 3, 331-336, <https://doi.org/10.1016/S0308-8146(02)00164-4> | [LINK](https://webbook.nist.gov/cgi/cbook.cgi?ID=C76222&Units=SI&Mask=2200) | DB-5 | 76-22-2 |
| 10.05 | terpinen-4-ol (syn. *p*-ment-1-en-4-ol) | 0.68 | 154 | C_10_H_18_O | 1189 | 1180 | 154, 136, 121, 111, 93, 91, 77, 71, 67 | Maia et al., Flavour Fragr. J., 2005, 20, 5, 474-477, <https://doi.org/10.1002/ffj.1499> | [LINK](https://webbook.nist.gov/cgi/cbook.cgi?Name=terpinen-4-ol&Units=SI&cMS=on&cGC=on) | DB-5MS | 877467-48-6 |
| 10.2 | α-terpineol | 1.63 | 154 | C_10_H_18_O | 1200 | 1190 | 155, 136, 121, 105, 93, 91, 77 67 | Hazzit et al., J. Agric. Food Chem., 2006, 54, 17, 6314-6321, <https://doi.org/10.1021/jf0606104> | [LINK](https://webbook.nist.gov/cgi/cbook.cgi?ID=C98555&Units=SI&Mask=2200#ref-13) | HP-5MS | 98-55-5 |
| 10.82 | carvone | 0.93 | 150 | C_10_H_14_O | 1257.6 | 1249 | 150, 135, 108, 93, 91, 79, 77, 54 | Baranauskiene et al., Flavour Fragr. J., 2005, 20, 5, 492-500, <https://doi.org/10.1002/ffj.1478> | [LINK](https://webbook.nist.gov/cgi/cbook.cgi?ID=C99490&Units=SI&Mask=2200) | BP-5 | 99-49-0 |

^a^Kovat’s index (KI) exp., experimental Kovat’s retention index calculated based on hydrocarbon mixture set as isothermic with logs (GsBP-5MS; 30m*0.25mm*0.50um; 15 °C/min to 300°C)

^b^Kovat’s index ref., reference Kovat’s retention index based on literature according to NIST Chemistry WebBook (SRD 69; July 2021).

^c^Reference literature and link to National Institute of Standards and Technology (NIST) is provided, https://webbook.nist.gov. The reference column condition: Kovats' RI non-polar column, temperature ramp (upon availability).

Bold value represents the major component detected in the volatile oil sample. Mw, molecular weight.
